# *PVT1* lincRNA signals an androgen‐dependent transcriptional activation program of oncogenes in prostate cancer cells

**DOI:** 10.1002/ijc.70417

**Published:** 2026-03-06

**Authors:** Maria Gabriela Berzoti‐Coelho, Fabio Nunes de Mello, Ana Carolina Tahira, Gabriel Nakanishi Fortes, Agatha Fischer‐Carvalho, Pedro Jardim Poli, Murilo Sena Amaral, Sergio Verjovski‐Almeida

**Affiliations:** ^1^ Cell Cycle Laboratory Instituto Butantan São Paulo Brazil; ^2^ Bioinformatics Interunits Graduate Program Universidade de São Paulo São Paulo Brazil; ^3^ Departamento de Bioquímica, Instituto de Química Universidade de São Paulo São Paulo Brazil

**Keywords:** androgen receptor signaling, epigenetic regulation, long non‐coding RNA, prostate cancer, *PVT1*

## Abstract

Prostate cancer is the most prevalent malignancy among men and is driven by multiple factors, including androgen signaling and its receptor. Long non‐coding RNAs, such as *PVT1*, play key roles in cancer, particularly by regulating gene expression. *PVT1* is upregulated in several cancer types and has been shown to interact with the androgen receptor in prostate cells. This study investigates how *PVT1* contributes to the prostate cancer phenotype under androgen stimulation. Knockdown of *PVT1* was achieved using CRISPR‐Cas13d in LNCaP prostate cancer cells subjected to androgen (R1881) or vehicle treatment. Cellular proliferation, invasion, and apoptosis rates were assessed, alongside RNA sequencing (RNA‐seq) to analyze genome‐wide transcriptomic changes. Six epigenetic marks—AR, EZH2, H3K4me1, H3K4me3, H3K27me3, and H3K27ac—were examined using CUT&RUN. *PVT1* knockdown led to a significant reduction in cell proliferation and an increase in apoptosis signaling. Oncogenes such as *MYC*, *AKT1*, *AKT2*, cyclins *CCNA2*, *CCNB1*, *CCNB2*, *CCNE1*, *CCNE2* and cyclin‐dependent kinases *CDK1* and *CDK4*, which were upregulated under androgen treatment, exhibited a significantly reduced expression following *PVT1* knockdown, thereby modulating cancer‐associated oncogenic pathways. Epigenetically, *PVT1* knockdown markedly decreased the occupancy of transcriptionally activating epigenetic marks—H3K4me1, H3K4me3, and H3K27ac—on oncogenes, regardless of androgen presence. Analysis of enriched transcription factors associated with the altered genes revealed a regulatory network linked to prostate cancer pathogenesis. *PVT1* drives a genome‐wide epigenetic reprogramming in prostate cells, underscoring the role of *PVT1* as a positive regulator of oncogenic pathways in prostate cancer and highlighting *PVT1*'s potential as a therapeutic target.

## What's New?

Unraveling the networks that regulate androgen receptor (AR) function is critical to better understanding prostate cancer development and progression. Of particular interest in this regard is the long non‐coding RNA *PVT1*, which regulates gene expression in cancer and is upregulated in prostate tumors. Here, the authors investigated the mechanistic role of *PVT1* in androgen‐driven prostate tumors using CRISPR‐Cas13d‐mediated *PVT1* knockdown combined with transcriptomic and epigenetic profiling. Experiments show that *PVT1* promotes genome‐wide epigenetic remodeling and enhances transcription of androgen‐dependent oncogenes. The findings identify *PVT1* as a key regulator of androgen‐driven tumorigenesis and a potential therapeutic target in prostate cancer.

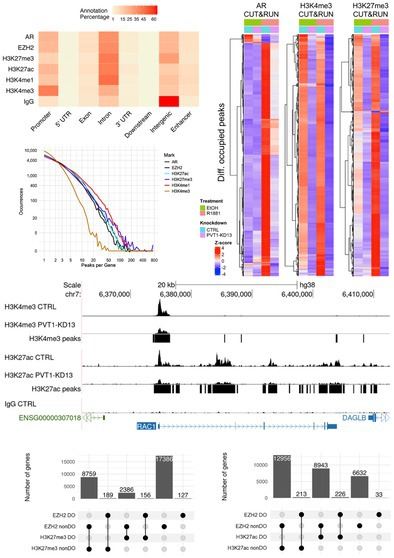

**Abbreviations**
AR: androgen receptor
CTRL: control
DO: differentially occupied genes
EtOH: ethanol
FDR: false discovery rate
gRNA: guide RNA
KD: knockdown
lincRNA: long intergenic non‐coding RNA
nonDO: non‐differentially occupied genes
PCa: prostate cancer
qPCR: quantitative real‐time PCR
RNA‐seq: RNA sequencing
RT: reverse transcription
TCGA‐PRAD: The Cancer Genome Atlas–Prostate Adenocarcinoma
WGCNA: Weighted Gene Co‐expression Network Analysis

## 1 INTRODUCTION

Prostate cancer (PCa) remains one of the leading causes of cancer‐related morbidity and mortality in men.^1^ Encompassing a multifactorial etiology, PCa is influenced by genetic predisposition along with age, lifestyle choices, and environmental factors.^2^ The androgen receptor (AR) has a pivotal role in PCa, acting as an oncogenic driver or tumor suppressor. This dual mechanism of action is mainly associated with its co‐regulators.^3^ Exploring the components underlying the AR‐associated factors is crucial to understanding PCa development.

The long intergenic non‐coding RNA (lincRNA) *PVT1* is an oncogene upregulated in various cancers,^4^ including PCa.^5^, ^6^ Its high expression is well correlated with poor prognosis,^5^ lower survival rate^7^ and resistance to chemotherapy.^8^ While the precise mechanisms through which *PVT1* contributes to oncogenesis are not fully understood, it has been implicated in key tumor‐related biological processes such as cell migration, invasion, proliferation, and apoptosis.^6^ LincRNA *PVT1* widely impacts several types of cancer through multi‐layered gene regulation.^9^

For instance, *PVT1* can bind to c‐Myc, a well‐known oncogene, and stabilize the protein, preventing its phosphorylation and degradation.^10^ In addition, *PVT1* promotes cellular proliferation and invasion in gastric cells by direct binding and stabilization of FOXM1.^11^ This mechanism is also observed in pancreatic and clear cell renal carcinoma through stabilization of HIF‐1α^12^ and HIF‐2α,^13^ respectively. Moreover, *PVT1* acts as a microRNA sponge in several tumors.^14^ In PCa, *PVT1* binds to miRNA‐186‐5p and positively regulates Twist1, promoting invasion and metastasis.^15^

*PVT1* epigenetically modulates chromatin through its capacity to recruit EZH2, which catalyzes trimethylation of lysine 27 of histone 3 (H3K27me3),^9^ to the promoter region of genes such as *LATS2* in non‐small cell lung cancer,^16^ p16 and p15 genes in gastric cancer,^17^ and to the miR‐195 promoter in cervical cancer.^18^ In PCa, our group showed that *PVT1* interacts with AR^19^ and EZH2^20^ in LNCaP cells, and that *PVT1* knockdown caused upregulation of hundreds of genes that were repressed by AR, pointing out that *PVT1* was involved in a genome‐wide AR‐dependent transcriptionally repressive regulation of tumor suppressor genes.^20^

Here, we further explored the mechanism by which *PVT1* acts in PCa under androgen treatment by mapping the genome‐wide changes in occupancy of six epigenetic marks in LNCaP cells upon *PVT1* knockdown. We found that *PVT1* mediates AR‐dependent transcriptional repression and activation and changes the genome‐wide deposition of histone marks to modulate both oncogenes and tumor suppressors. Global expression and epigenetic changes pointed to a well‐defined gene network involved in cancer development, highlighting *PVT1*'s oncogenic role in PCa.

## 2 MATERIALS AND METHODS

### 2.1 Cell culture

Androgen‐dependent LNCaP clone FGC Prostate Carcinoma Human (ATCC CRL1740, USA) (RRID:CVCL_1379) was used. The cell line has been authenticated using STR profiling within the last 3 years, and all experiments were performed with mycoplasma‐free cells. Cells were cultured as detailed in the Data S1 (Methods).

### 2.2 *PVT1* knockdown

Knockdown of *PVT1* was carried out via CRISPR‐Cas13d system, using each of two distinct guide RNA (gRNA) sequences targeting *PVT1*, designated PVT1‐KD13 and PVT1‐KD17 (Table S1). The two gRNAs were cloned into lentiviral vector pLentiRNAGuide_002 (Addgene #138151, USA), and empty vector was used as negative control. Lentiviral pLentiRNACRISPR_007 plasmid (Addgene #138149) was used for doxycycline‐inducible RfxCas13d expression. See Data S1 (Methods) for lentiviral construction and for LNCaP transduction protocol.

To induce *PVT1* knockdown, LNCaP transduced cells were treated with 2 μg/mL doxycycline hydrochloride (Sigma‐Aldrich, USA) for 96 h, with the medium refreshed and doxycycline replaced at 48 h of culture.

### 2.3 Cells' treatment with androgen analog R1881

PVT1‐KD or CTRL LNCaP cells were seeded in 175 cm^2^ flasks at a density of 1 × 10^7^ cells/flask. After 48 h, cells were washed once with PBS (Gibco) and cultured in fresh medium supplemented with 10% charcoal‐stripped fetal bovine serum (Gibco) to eliminate hormonal activity. Cells were maintained in hormone‐depleted medium for 48 h, with medium replacement after 24 h. Subsequently, cells were treated with 10 nM R1881 (Methyltrienolone; Sigma‐Aldrich) or the corresponding volume of vehicle ethanol (EtOH) for 48 h, with a fresh dose added after 24 h. Doxycycline was replenished at 2 μg/mL with every medium change, from initial plating through the end of treatment.

### 2.4 RNA extraction, cDNA synthesis, and RT‐qPCR

Briefly, RNA was extracted using RNeasy Micro Kit (Qiagen, USA) following the manufacturer's protocol. For cDNA synthesis, 500 ng to 1 μg of total RNA was used in reverse transcription (RT) reactions with the SuperScript IV First‐Strand System (Invitrogen, USA) and random hexamer primers, following the manufacturer's instructions. Quantitative real‐time PCR (qPCR) was performed using gene‐specific primers for the target genes (Table S2). See the detailed protocol in Data S1 (Methods).

### 2.5 Proliferation assays

Trypan blue exclusion assays were conducted using 3.5 × 10^5^ LNCaP cells/well (CTRL or PVT1‐KD13/PVT1‐KD17), and cell proliferation was evaluated for 5 days at 24 h intervals. To ensure accuracy, six different cell‐to‐trypan blue dilutions (all at a 1:1 ratio) were prepared for each condition. See the detailed protocol in Data S1 (Methods).

### 2.6 Invasion assays

Fluoroblok transwell inserts (Becton Dickinson, USA) with 8 μm pores pre‐coated with Geltrex (Gibco) were used. CTRL or PVT1‐KD13/PVT1‐KD17 LNCaP cells (1.7 × 10^5^ cells) suspended in serum‐free medium in the Geltrex‐coated inserts were exposed to medium supplemented with 20% fetal bovine serum as a chemoattractant in the lower chamber, beneath the insert. Cells were incubated for 48 h to allow for invasion. See the detailed protocol in Data S1 (Methods).

### 2.7 Apoptosis assays

To induce apoptosis, CTRL or PVT1‐KD13/PVT1‐KD17 LNCaP cells were treated with staurosporine. A total of 5 × 10^6^ cells/condition was seeded in 10 cm^2^ culture dishes. After 48 h, cells were treated overnight with 1 μg/mL staurosporine, or an equivalent volume of vehicle (DMSO), followed by washing once with PBS and lysis in RIPA buffer supplemented with a protease inhibitor cocktail (PIC; Sigma‐Aldrich). Protein concentration determination and western blotting assays are described in Data S1 (Methods).

### 2.8 Cut&run

The CUT&RUN assays^21^ were run in biological duplicate, following the manufacturer's protocol for the CUT&RUN Kit (Cell Signaling Technology, #86652, USA). For each experimental condition—negative control (IgG), input, four histone mark targets (H3K27ac, H3K4me1, H3K4me3, H3K27me3), EZH2 and AR—100,000 CTRL or PVT1‐KD13 live cells, treated with androgen analog (R1881) or vehicle (ethanol, EtOH), were used. Antibody volumes were applied according to the manufacturer's recommendations for each target (Table S3). Input samples were generated by sonication using the Covaris S2 ultrasonicator, with parameters detailed in Table S4. DNA extraction and library construction are detailed in Data S1 (Methods).

DNA libraries were multiplexed and sequenced on a DNBseq (BGI Genomics, China), with 50 bp paired‐end sequencing for CUT&RUN samples and 100 bp paired‐end sequencing for input samples, and ~ 20 million reads/sample. CUT&RUN sequencing coverage and sequence quality statistics for each sample are summarized in Table S5, and data analyses are detailed in Data S1 (Methods), including peak counts normalization with the CUT&RUN Greenlist.^22^

### 2.9 RNA‐seq

Three biological replicates of CTRL or PVT1‐KD13 cells, treated with either androgen analog (R1881) or vehicle (EtOH), underwent RNA extraction as described above and treated with Turbo DNase (Invitrogen) according to the manufacturer's instructions. Sample quality was then assessed using a Bioanalyzer system with the RNA 6000 Pico Kit (Agilent, USA). Samples' stranded cDNA libraries preparation was performed on‐site by BGI Genomics, and libraries were sequenced on the DNBseq platform, with 150 bp paired‐end sequencing and ~ 30 million reads/sample. RNA‐seq sequencing coverage and quality statistics for each sample are summarized in Table S5, and data analyses are detailed in Data S1 (Methods).

### 2.10 Statistical analysis

The non‐parametric Mann–Whitney test was used, with *p*‐values <0.05 considered statistically significant for qPCR analysis, invasion and proliferation assay. For CUT&RUN analysis, differentially occupied (DO) sites were considered significant with *p*‐adjusted values ≤0.05. For RNA‐seq, differentially expressed genes were considered significant with *p*‐adjusted values ≤0.01. See the detailed analysis in Data S1 (Methods).

## 3 RESULTS

### 3.1 *PVT1* expression correlates with prostate cancer clinical samples displaying gene expression programs involved with cell growth, survival, and metabolism

**FIGURE 1:**
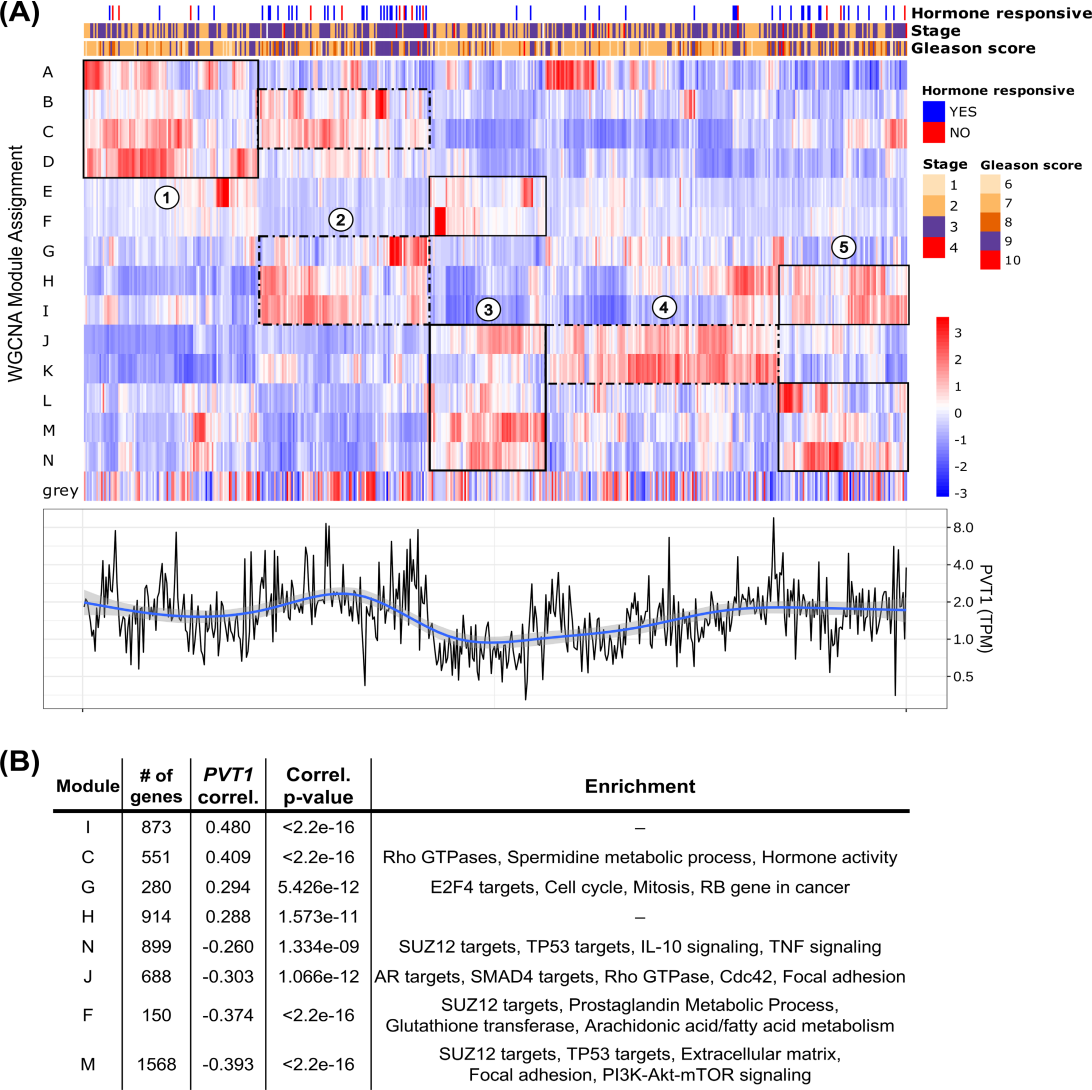
Clinical prostate cancer samples show diverse gene expression profiles. (A) Upper panel: Clustered RNA‐seq eigengene expression profile of 528 prostate cancer clinical samples from TCGA. WGCNA‐identified 14 gene modules (A to N on y‐axis); genes not clustered in any co‐expression module are pooled on the bottom grey module. Samples on x‐axis and identified at the top by reported cancer stage, tumor Gleason score, and hormone treatment responsiveness (if available). Expression z‐scores calculated by row (z‐score color scale at right); columns and rows ordered by complete linkage hierarchical clustering of correlation. Circled numbers highlight the 5 groups of patient samples, each with a distinct pattern of modules eigengene expression; black boxes highlight the modules with higher eigengene expression within each group. Lower panel: Normalized *PVT1* expression per sample shown with the same x‐axis. (B) Functional enrichment of WGCNA modules significantly (*p* < 0.01) correlated with *PVT1* expression (*PVT1* gene itself is in module I).

We previously showed that high levels of *PVT1* in prostate cancer patient samples from the TCGA‐PRAD cohort (https://portal.gdc.cancer.gov) are a statistically significant predictor of a shorter disease‐free survival^20^; additionally, we demonstrated that knocking down *PVT1* lncRNA in androgen‐stimulated LNCaP cells significantly changes the expression of hundreds of genes.^20^ This prompted us to reinvestigate these prostate cancer patient samples, and to look for the genes that show correlated expression with *PVT1*. To explore this, we re‐analyzed the RNA‐seq data of the 528 PCa samples from 497 patients in the TCGA‐PRAD cohort using Weighted Gene Co‐expression Network Analysis (WGCNA) and identified 14 distinct modules of co‐expressed genes, labeled A through N (Figure 1A, upper panel, Table S6). These samples were annotated based on reported cancer stage, tumor Gleason score, and hormone treatment responsiveness (Figure 1A, top). We found no clear correlation between the modules and clinical features, underscoring the heterogeneity among samples and PCa phenotypes.

Of interest, we observed that patient samples did cluster within five groups (Figure 1A, upper panel circled numbers), each with a distinct pattern of higher gene expression at different modules: group 1, modules A to D; group 2, modules B, C, G, H and I; group 3, modules E, F, and J to N; group 4, modules J and K; group 5, modules H, I, and L to N (Figure 1A, upper panel black boxes). *PVT1* expression levels varied across the samples with a pattern that closely followed the five distinct groups (Figure 1A, lower panel).

We identified eight modules that were significantly correlated with *PVT1* expression: modules I, C, G, and H positively correlated with *PVT1*, while modules N, J, F, and M showed a negative correlation (Figure 1B). Pathway enrichment analysis revealed that most modules were enriched in cancer‐related pathways crucial for cell growth, survival, and metabolism. Interestingly, no pathway enrichment was found in module I, where *PVT1* is clustered (Figure 1B and Table S7).

### 3.2 *PVT1* knockdown impacts cell proliferation and apoptosis

**FIGURE 2:**
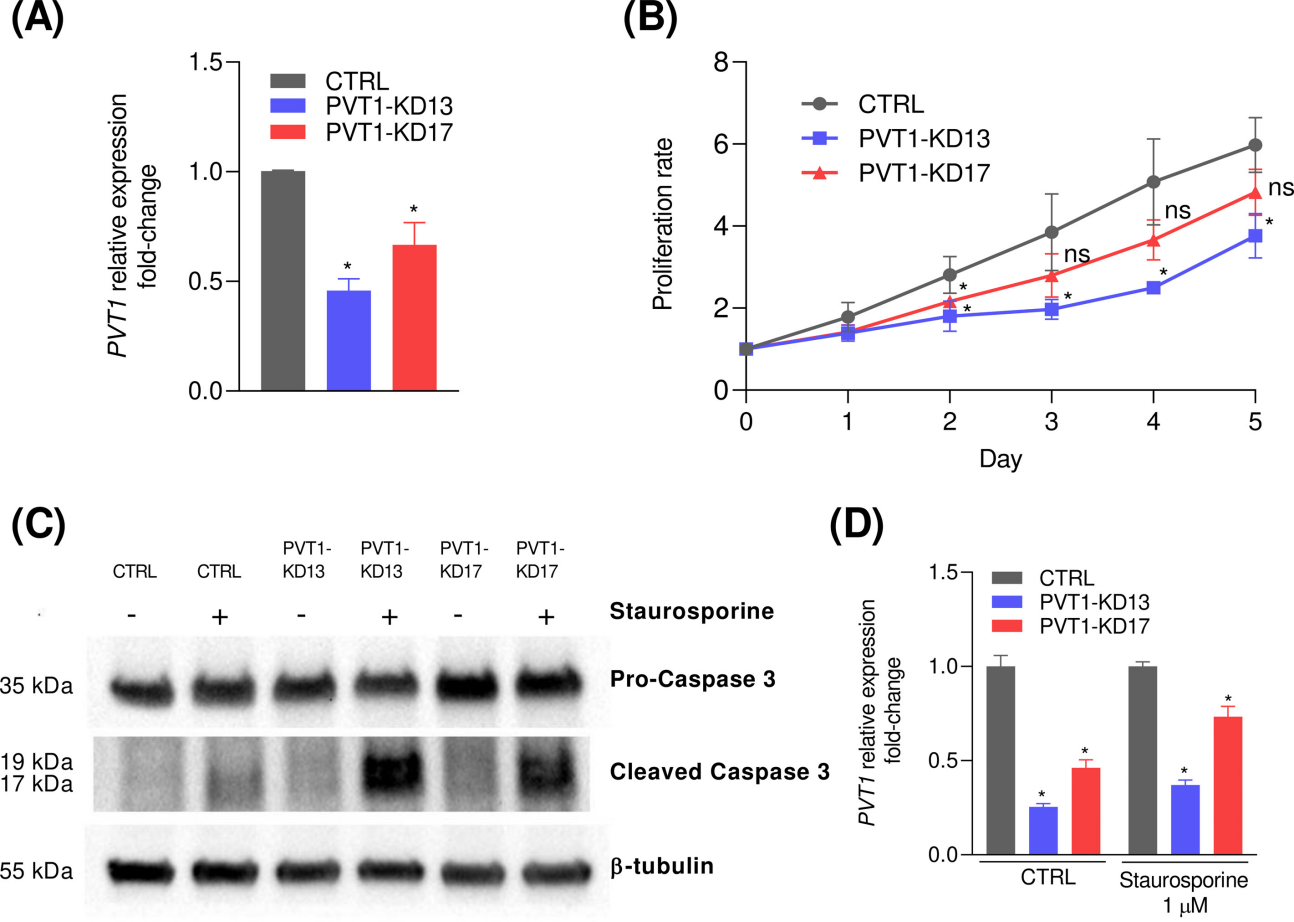
Knockdown of *PVT1* impairs cell survival in prostate cancer. (A) *PVT1* expression measured by RT‐qPCR in LNCaP cells under control (CTRL) and *PVT1* knockdown conditions obtained via CRISPR‐Cas13d inducible system, using two different guide RNAs targeting *PVT1* (PVT1‐KD13 or PVT1‐KD17); *n* = 4, *p < 0.05. (B) Proliferation of LNCaP cells measured by Trypan Blue Exclusion Assay. Proliferation time course in CTRL and PVT1‐KD13/PVT1‐KD17conditions, normalized to day 0; *n* = 4, *p < 0.05. (C) Representative results of Pro‐Caspase 3 and cleaved Caspase 3 western blotting after 96 h of *PVT1* knockdown and overnight staurosporine (Stauro) treatment. Since cleaved Caspase 3 abundance is typically low, staurosporine was used to enhance its accumulation. (D) Relative *PVT1* expression measured by RT‐qPCR in aliquots from samples of the caspase assays of panel (C). *p <0.05.

To further explore *PVT1* effects in the PCa oncogenic scenario, we performed *PVT1* knockdown (KD) in LNCaP cells using a doxycycline‐inducible CRISPR‐Cas13d system and two different *PVT1* RNA‐targeting guides (PVT1‐KD13 or PVT1‐KD17) (Figure 2A). Aligned with previous findings establishing an oncogenic role for *PVT1*,^4^ proliferation of LNCaP cells was significantly reduced following *PVT1* KD (Figure 2B). The proliferation rate correlates with *PVT1* expression levels: lower *PVT1* expression (Figure 2A, blue bar) leads to a reduced proliferation rate (Figure 2B, blue line).

Additionally, the apoptosis rate was affected by *PVT1* KD. After treating LNCaP cells with staurosporine to induce apoptosis, PVT1‐KD13/PVT1‐KD17 cells exhibited higher levels of cleaved caspase‐3 compared with CTRL cells or with KD cells in the absence of staurosporine (Figures 2C and S1). This indicates that *PVT1* may play a protective role against apoptosis, whilst *PVT1* KD (Figure 2D) sensitizes LNCaP cells to apoptosis (Figure 2C). We also evaluated the impact of *PVT1* knockdown on the invasion potential of LNCaP cells (Figure S2). As expected, due to LNCaP's low invasive potential,^23^ no differences were observed between PVT1‐KD13/PVT1‐KD17 and CTRL cells (Figure S2).

### 3.3 *PVT1* knockdown alters the response of PCa cells to androgen by impacting specific pathways and transcription factors

**FIGURE 3:**
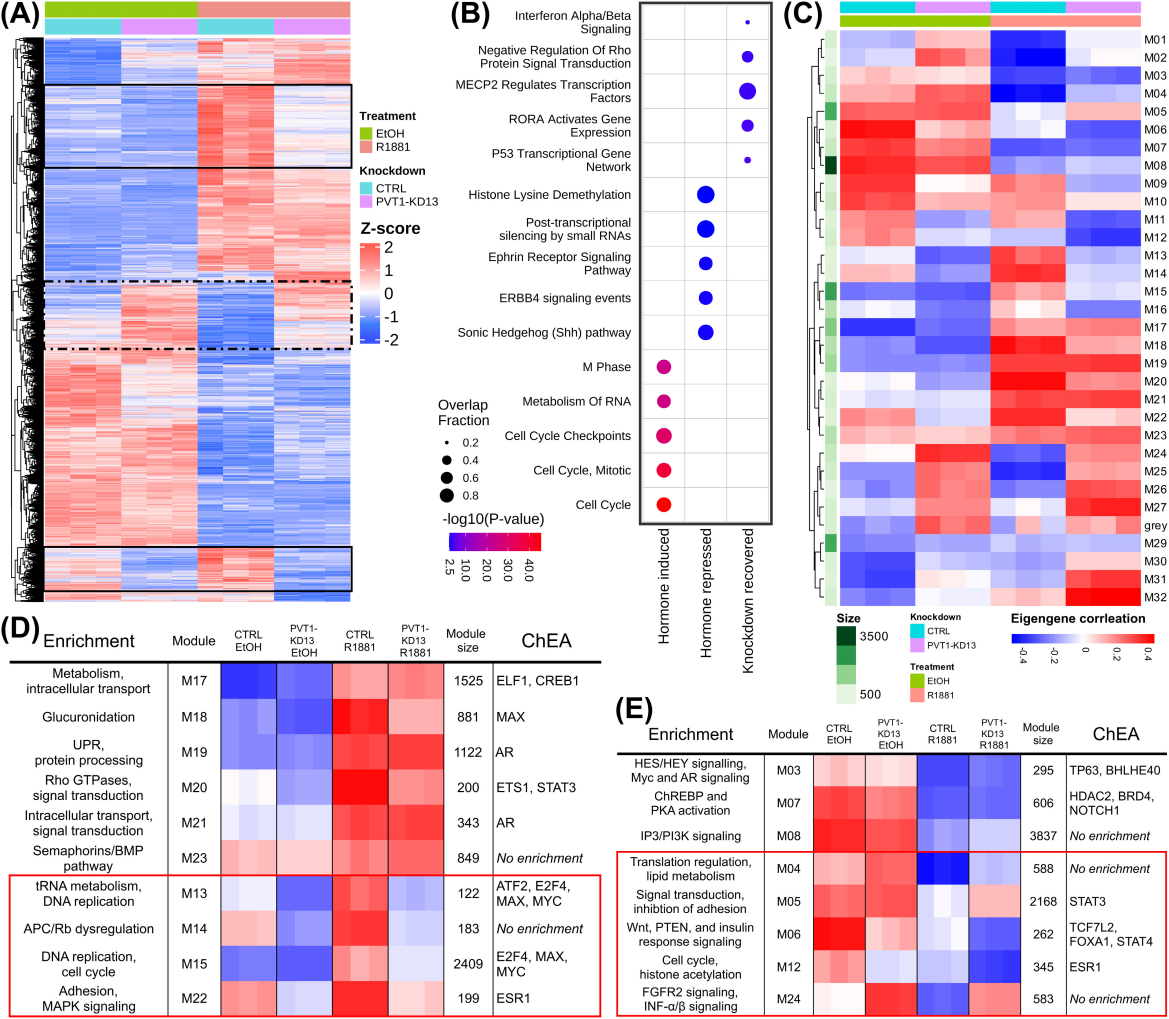
Knockdown of *PVT1* changes the response of prostate cancer cells to androgen. (A) Expression heatmap of genes significantly different upon either hormone treatment (R1881) or PVT1‐KD13 of hormone‐treated cells () compared with CTRL cells without knockdown, identified by RNA‐seq. Black solid boxes highlight genes whose expression was upregulated by androgen treatment and reversed upon *PVT1* knockdown; black dashed box highlights genes that were repressed by androgen treatment and recovered upon *PVT1* knockdown. Z‐scores calculated by row; genes (y‐axis) ordered by complete linkage hierarchical clustering of correlation, three biological replicates per condition (x‐axis). (B) Functional enrichment of genes upregulated by hormone (Hormone induced), downregulated by hormone (Hormone repressed), or downregulated in the presence of hormone and rescued by knockdown of *PVT1* (Knockdown recovered), as indicated in the x‐axis. Enrichment calculated by hypergeometric test based on several pathway databases (see Section 2); *p* < 0.003 shown, five most significant enrichment terms selected per gene set. (C) Eigengene correlation heatmap of WGCNA clusters from RNA‐seq data. Correlation color scale at bottom. Gene modules (y‐axis) ordered by complete linkage hierarchical clustering of correlation; three replicates per condition (x‐axis), gene count per module shown on the left in a green scale (size). (D) Functional enrichment of WGCNA modules that showed increased expression upon androgen treatment; modules whose expression was affected by *PVT1* knockdown are surrounded by the red box. On the leftmost column, enrichment of combined Gene Ontology and Reactome terms; on the rightmost column, ChIP‐X Enrichment Analysis (ChEA) for putative regulatory transcription factors of query gene‐sets. (E) Functional enrichment of WGCNA modules as in (D), but for modules that showed decreased expression upon androgen treatment; modules whose expression was affected by *PVT1* knockdown are surrounded by the red box.

Based on our previous findings that *PVT1* regulates genome‐wide gene expression in PCa cells in an AR‐dependent manner,^19^, ^20^ we performed bulk RNA‐Seq (Figure 3A) on control (CTRL) and PVT1‐KD13 LNCaP cells, treated with androgen analog R1881 or the vehicle ethanol (EtOH) (Figure S3). PVT1‐KD13 in androgen‐stimulated LNCaP cells significantly upregulated and downregulated hundreds of genes (Figure 3A and Table S8), confirming and extending our previous results.^20^ Of note, we observed genes whose expression was upregulated by androgen treatment and subsequently repressed upon PVT1‐KD13 (Figure 3A, solid black boxes, Table S8); among them, several oncogenes were upregulated, as described further below.

When analyzing CTRL cells (without PVT1‐KD13), hypergeometric enrichment analysis based on several pathway databases revealed that hormone‐induced genes were associated with hormone‐dependent functions in LNCaP cells, such as metabolism and cell cycle regulation (Figure 3B and Table S9).

Interestingly, hormone‐repressed genes revealed pathways involved in embryonic development and axonal function (including Shh, ErbB4, and Ephrin pathways) (Figure 3B and Table S9). Conversely, these pathways play important roles in processes like proliferation, differentiation, migration, and apoptosis, all of which are described in cancer.^24^, ^25^

Additionally, we again observed genes whose expression was repressed by androgen treatment and subsequently recovered upon PVT1KD13 (Figure 3A, dashed black box and 3B).^20^ These genes showed enrichment in pathways affected by the transcription factors (TFs) regulatory proteins MECP2 and RORα (Figure 3B and Table S9), suggesting the role of *PVT1* in epigenomic regulation.^26^

Using WGCNA, we identified 32 modules, labeled M01 to M32 (Figure 3C and Table S10) enriched in specific pathways and TFs associated with hormone‐induced modules (Figure 3D) and hormone‐repressed modules (Figure 3E). Notably, we identified modules affected by *PVT1* knockdown, distinguishing the hormone‐only response from the *PVT1*‐mediated response.

In hormone‐induced modules, *PVT1* appeared to act in conjunction with androgen for the upregulation induced by hormone treatment, specifically in modules M13, M14, M15, and M22 (Figure 3D, red box), where importantly, PVT1‐KD13 reversed the androgen‐induced genes upregulation. Enriched pathways involved with these modules are linked to critical cellular processes related to oncogenesis, such as DNA replication (M13), cell cycle regulation (M15), cell growth, and proliferation, including APC/Rb tumor suppressor dysregulation (M14) and MAPK signaling pathway (M22), further supporting the oncogenic role of *PVT1* (Figure 3D).

Notably, module M15 was significantly enriched in cell cycle related oncogenes such as *MYC*, *AKT1*, *AKT2*, and cyclins *CCNA2*, *CCNB1*, *CCNB2*, *CCNE1*, *CCNE2*, cyclin‐dependent kinases *CDK1* and *CDK4*, as well as cell cycle‐promoting phosphatases *CDC25A*, *CDC25B* and *CDC25C*, all of which were upregulated by hormone treatment and subsequently downregulated by *PVT1* knockdown (Table S8). Transcription factors ATF2, E2F4, MAX, MYC and ESR1 are enriched in the promoters of genes belonging to these modules (Figure 3D).

In hormone‐repressed modules (Figure 3E), the *PVT1*‐mediated response was dual (Figure 3E, red box). In modules M04, M05, and M24, gene expression downregulation induced by hormone was attenuated by *PVT1*KD13, showing a synergistic effect of androgen and *PVT1* in affecting pathways related to translation regulation, lipid metabolism, inhibition of adhesion, FGFR2, and interferon alpha and beta signaling. On the contrary, in modules M06 and M12, downregulation induced by hormone was accentuated by *PVT1* KD, affecting pathways related to Wnt, PTEN and insulin response signaling, cell cycle and histone acetylation, and involving genes enriched with transcription factors TCF7L2, FOXA1, STAT4 and ESR1 at their promoters.

### 3.4 *PVT1* knockdown leads to a global reorganization of the epigenetic landscape in PCa

To gain a deeper insight into the epigenetic program regulation exerted by *PVT1*, we treated LNCaP CTRL or PVT1‐KD13 cells with either R1881 or vehicle (EtOH) (Figure S4) and conducted CUT&RUN assays. We mapped histone modifications (H3K27ac, H3K4me1, H3K4me3, and H3K27me3), as well as AR and EZH2 occupancy at the annotated gene *loci* along the genome.

**FIGURE 4:**
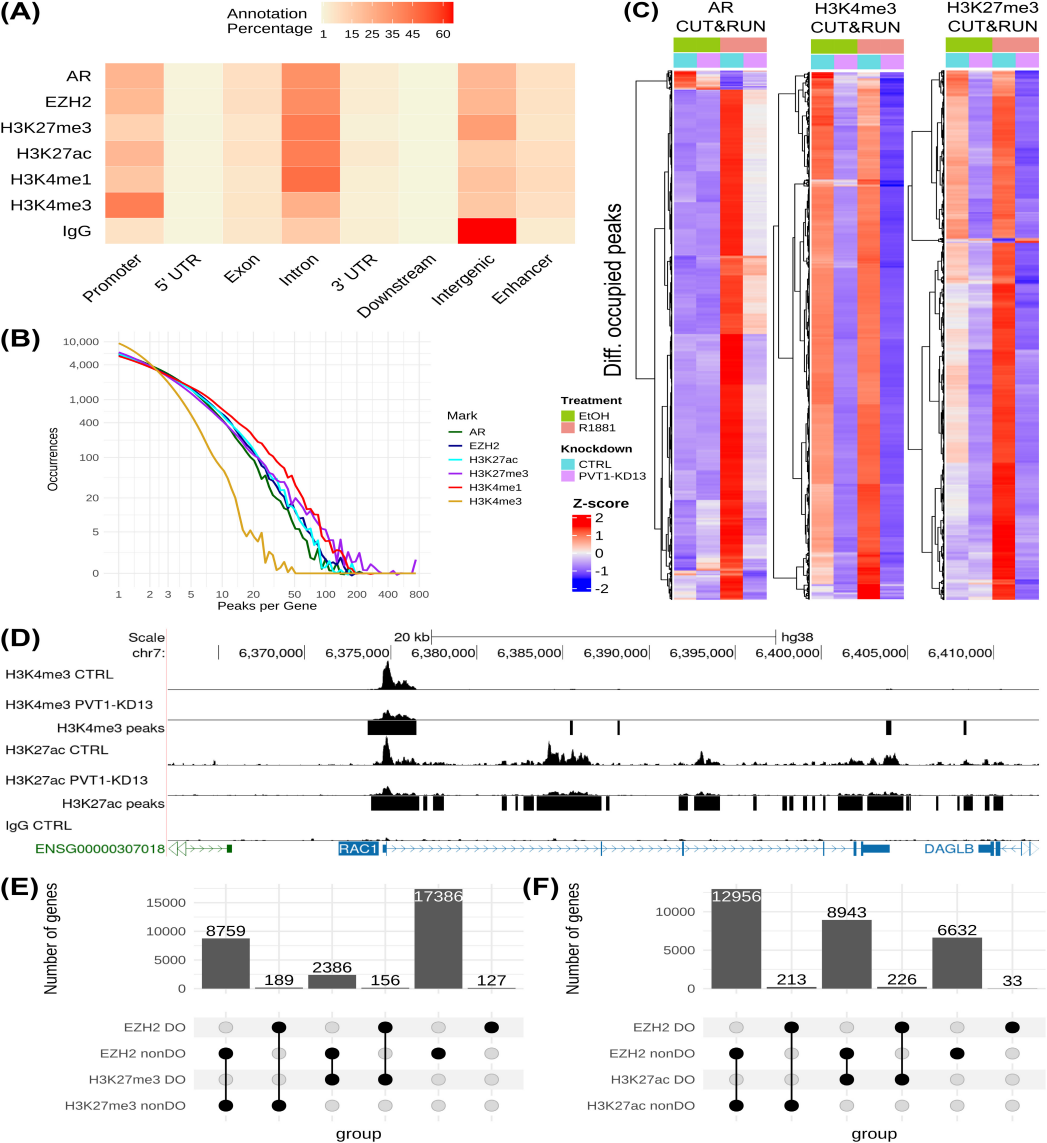
Knockdown of *PVT1* significantly reshapes the epigenetic landscape of prostate cancer cells. (A) Heatmap of CUT&RUN peak distribution along gene body elements, shown as percent of total peaks per antibody. “Promoter” considered up to 5 kb upstream of TSS, “downstream” considered up to 300 bp downstream of TES, “enhancer” annotated based on known distal enhancers (see Section 2). (B) Histogram of distinct CUT&RUN peaks called per gene for each antibody used. (C) Heatmaps of normalized epigenetic mark occupancy for statistically significant peaks at each gene (rows, y‐axis). Peak abundance for each epigenetic mark at each gene (y‐axis), shown as average of two replicates, ordered by complete linkage hierarchical clustering of correlation; z‐scores calculated by rows; assay conditions (x‐axis) are indicated by the color codes at top and the color‐coded legends at left. Results for anti‐AR, anti‐H3K4me3 and anti‐H3K27me3 CUT&RUN shown as indicated at the top. (D) Representative CUT&RUN occupancy tracks over the *RAC1* gene body locus, comparing accumulation of H3K4me3 and H3K27ac between CTRL and PVT1‐KD13 conditions in hormone treated cells. Occupancy accumulations are normalized (see Section 2) and shown as average of two replicates; tracks y‐axes scaled to respective controls; significantly enriched peaks are marked as black blocks in the third, lowermost track per antibody. (E) Upset plot showing the number of genes with EZH2 and/or H3K27me3 deposition, discriminating between differentially occupied (DO) or non‐differentially occupied (nonDO) genes upon *PVT1* knockdown. (F) Upset plot as in (E), but for EZH2 and/or H3K27ac deposition.

Peak distribution primarily occurred in intronic regions, followed by promoter and intergenic regions. H3K27me3 showed the highest occupancy in introns and intergenic regions, while promoter regions were predominantly occupied by H3K4me3 (Figure 4A). Across all marks, the distribution of peaks/gene was highly consistent, showing a concentrated presence in a moderate number of genes. Notably, H3K4me3 exhibited the smaller density of peaks/gene (Figure 4B).

R1881 treatment of LNCaP cells significantly increased AR occupancy (Figure 4C, left panel), with no noticeable shift in H3K4me3 occupancy (Figure 4D, middle panel). The hormonal response also enhanced H3K27me3 occupancy (Figure 4C, right panel), though to a lesser extent compared with AR occupancy. Knockdown of *PVT1* generally reduced occupancy of all marks, both in the presence and absence of hormone treatment (Figure 4C, all panels, Table S11). As a typical example, in the *RAC1 locus* (Figure 4D), for both H3K4me3 and H3K27ac transcriptional activation marks, the occupancy under hormone stimulation was diminished in PVT1‐KD13 cells compared with CTRL cells (Figure 4D). *RAC1* is a member of the RAC subfamily, extensively studied among the 20 subfamilies of Rho GTPases involved in cancer progression and dissemination.^27^

We previously demonstrated by native RIP‐qPCR that *PVT1* is associated with EZH2 in LNCaP cells, a subunit of PRC2.^20^ In this context, we analyzed PVT1‐KD13 cells compared with CTRL cells, both under hormone stimulation, and identified contrasting patterns regarding EZH2, H3K27ac, and H3K27me3 occupancy upon PVT1‐KD13 (Figure 4E,F). We classified the genomic *loci* that significantly changed following PVT1‐KD13 as differentially occupied (DO), while those that remained unchanged as non‐differentially occupied (nonDO) (Table S12). Of note, upon PVT1‐KD13, 28,531 (98.4%) out of 29,003 genes had their *loci* non‐differentially occupied by EZH2 (EZH2 nonDO; Figure 4E), while only 472 genes (1.6%) had their *loci* differentially occupied by EZH2 (EZH2 DO; Figure 4E and Table S12).

We found that upon PVT1‐KD13, 2386 genes were DO by H3K27me3, while their occupancy by EZH2 was not affected (Figure 4E). These observations suggest that the loss of H3K27me3 deposition at these genes following PVT1‐KD13 is not due to decreased EZH2 occupancy at their *loci*.

Concerning H3K27ac, there is a decrease of H3K27ac concomitantly with a persistent EZH2 occupancy; 8943 genes were DO by H3K27ac upon PVT1‐KD13 with no affected occupancy by EZH2 (Figure 4F and Table S12), suggesting that *PVT1* can affect in a genome‐wide manner the acetylation of H3K27 through a mechanism that warrants further characterization. We also identified 226 genes DO by both EZH2 and H3K27ac.

### 3.5 *PVT1* affects deposition of gene‐activating histone modifications in genes associated with pro‐oncogenic functions in response to androgen stimulation

**FIGURE 5:**
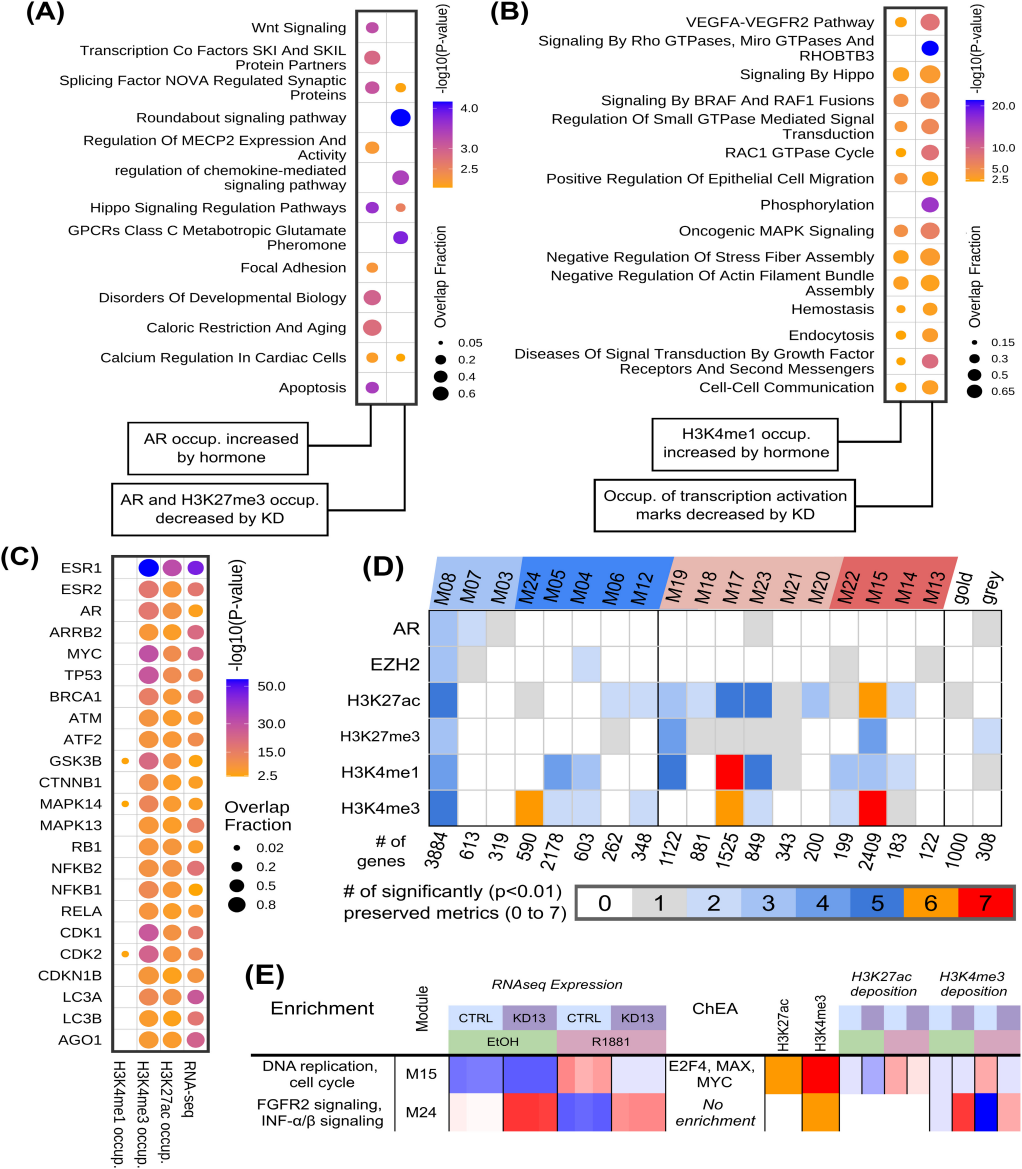
*PVT1* induces epigenetic changes in key signaling pathways. (A) Functional enrichment of genes with hormone‐driven increase of AR occupancy (left column) and knockdown‐driven depletion of both AR and H3K27me3 occupancy in the presence of hormone (right column). Enrichment calculated by hypergeometric test based on several pathway databases (see Section 2); *p* < 0.01 shown, most significant enrichment terms selected; list of genes in Table S13. (B) Functional enrichment of genes with hormone‐driven increase of H3K4me1 occupancy (left column) and knockdown‐driven loss of occupancy of all three transcription activation marks (H3K4me1, H3K4me3, H3K27ac) (right column). Enrichment calculated by hypergeometric test based on several pathway databases (see Section 2); *p* < 0.003 shown, and most significantly enriched terms selected; list of genes in Table S13. (C) Enrichment of reported protein–protein interaction partners of genes with the indicated decrease in histone marks occupancy, or decrease in gene expression by RNA‐seq, upon *PVT1* knockdown in the presence of hormone. Enrichment calculated by hypergeometric test; *p* < 0.04 shown, for a selected set of genes with enriched partners (see Data S1 [Methods]); list of partners in Table S14. (D) Preservation of WGCNA co‐expression modules projected onto the CUT&RUN histone marks data (see Section 2), where projections with 6 or 7 significantly preserved metrics (*p* < 0.01) suggest that the pattern of occupancy for that histone mark of the genes in that module, observed by CUT&RUN, follows the co‐expression observed by RNA‐seq (see Section 2). At the top, modules with a loss of expression upon hormone treatment are shaded blue, modules with an increase of expression are shaded red, and modules with *PVT1* knockdown‐dependent changes are shaded in darker blue or red. (E) Functional enrichment of the modules with significant preservation in CUT&RUN data. Enrichment of combined Gene Ontology and Reactome terms are shown on the left column, Modules and modules Expression Eigengenes are on the next five columns. ChIP‐X Enrichment Analysis (ChEA) for transcription factors at the promoters are shown on the center right column, and module preservation (as in D) shown on the two columns to the right of ChEA. The eight columns on the right show the changes in the histone marks deposition for genes in the modules, under the assay conditions indicated by the color code at the top (similar to the color code for RNAseq expression).

Functional enrichment analysis of genes with increased AR occupancy under hormone stimulation highlighted genes linked to hormone‐dependent functions involved in metabolism and proliferation (Figure 5A, left column, Table S13).

In contrast, hormone stimulation combined with PVT1‐KD13 decreased AR and H3K27me3 occupancy in genes involved with significant signaling pathways (Figure 5A, right column, Table S13); particularly, the Roundabout pathway, known for roles in angiogenesis, cell migration, and metastasis, acting either as an oncogene or a tumor suppressor.^28^

H3K4me1 was the only histone modification directly influenced by hormone stimulation. Functional enrichment analysis of genes with significant increase in H3K4me1 occupancy highlighted cancer‐related pathways and TFs, including MAPK signaling and BRAF/RAF1 fusions (Figure 5B, left column, Table S14).

Functional enrichment analysis of genes affected by H3K4me1, H3K27ac, and H3K4me3 transcriptional activation marks identified classical cancer‐related pathways, including MAPK signaling and VEGFA‐VEGFR2. Notably, the enrichment was also significant for genes associated with the Rho GTPases and Hippo pathways (Figure 5B, right column, Table S14), both implicated in cancer by influencing proliferation, adhesion, and migration.^29^, ^30^

Hypergeometric test identified significant enrichment of protein–protein interaction (PPI) partners whose genes showed a decrease in H3K4me1, H3K4me3, and H3K27ac occupancy or a decrease in gene expression following PVT1‐KD13 (Figure 5C and Table S14). Among the genes with significantly enriched protein–protein interacting partners with epigenetic changes, there were cell cycle regulators (*CDK1*, *CDK2*, *RB1*, *CDKN1B*), nuclear hormone receptors (*ESR1*, *ESR2*, *AR*), transcription factors (*MYC*, *ATF2*, *NFKB1*, *NFKB2*, *RELA*, *STAT3*), signaling molecules (*MAPK14*, *MAPK13*, *ARRB2*, *GSK3β*), and key mediators of cellular processes (*TP53*, *BRCA1*, *ATM*, *CTNNB1*, *MAP1LC3A*, *MAP1LC3B*) (Figure 5C).

Next, we evaluated whether the WGCNA RNA‐seq patterns of changes upon PVT1‐KD13 (Figure 3C) were preserved in the CUT&RUN data. To assess whether the genes within each module exhibited patterns of histone mark enrichment consistent with the patterns of RNA‐seq expression, we tested seven statistical preservation metrics (Figure 5D), according to Ritchie and collaborators^31^ (see Data S1 [Methods]).

Three modules were found preserved: M15, M17, and M24 (Figure 5D). The genes in M17 showed hormone‐driven increased expression, unaffected by *PVT1*, and regulated by H3K4me1 and H3K4me3 occupancy (Figure 5D). Modules M15 and M24 were both influenced by *PVT1* (Figure 5D). While genes in M24, which were regulated by H3K4me3 occupancy, exhibited decreased expression upon hormone treatment, genes in M15, impacted by both H3K4me3 and H3K27ac, showed increased expression under hormonal influence with reduced expression upon PVT1‐KD13 (Figure 5D).

The modules regulated by *PVT1* are linked to key cellular processes and pathways involved in oncogenesis: M24 genes are associated with FGFR2 and INFα/β signaling, while M15 genes are connected to DNA replication and the cell cycle and are associated with transcription factors E2F4, MYC, and MAX (Figure 5E).

### 3.6 *PVT1* in androgen‐stimulated LNCaP cells modulates a gene network involved in PCa

**FIGURE 6:**
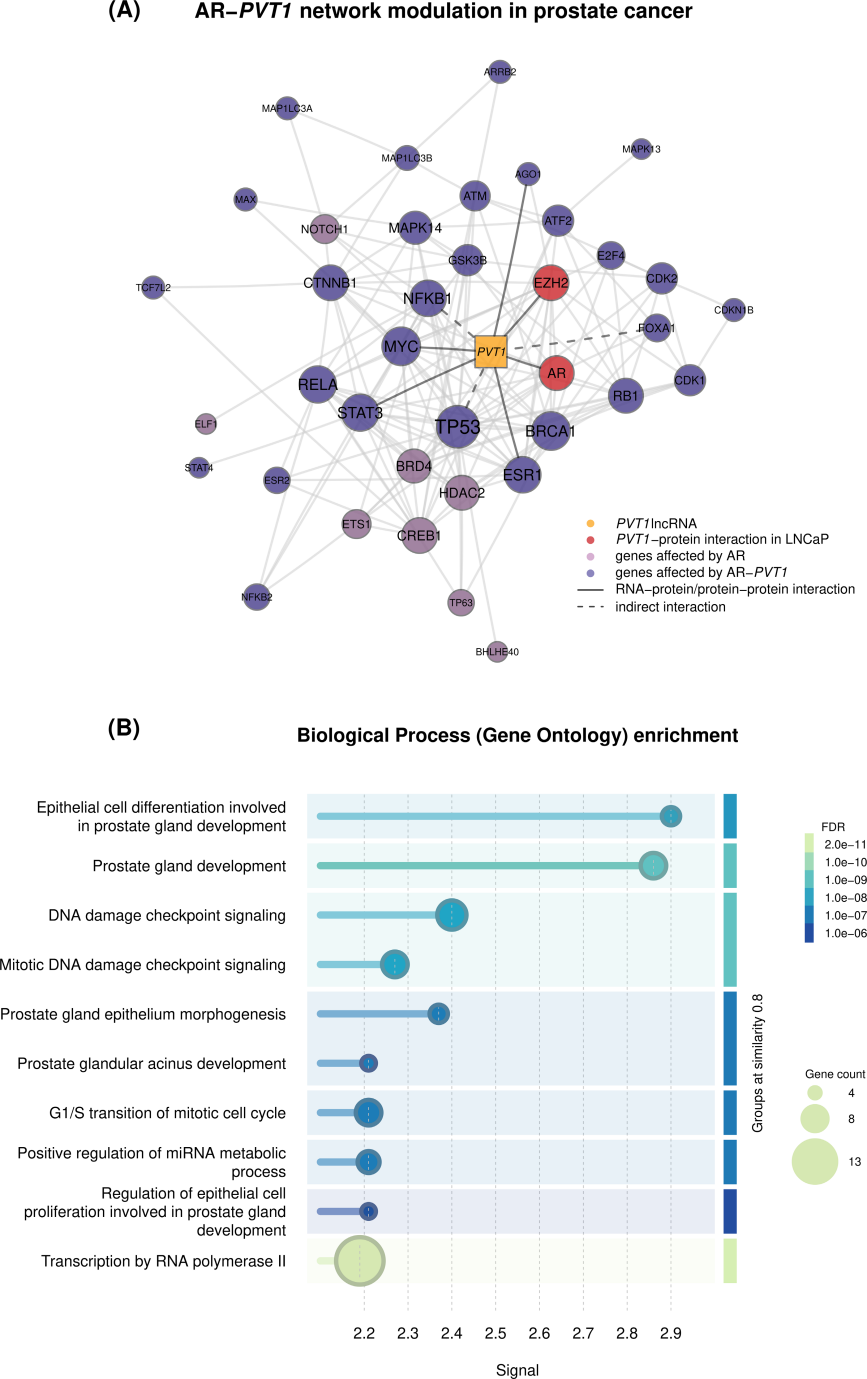
AR‐*PVT1* network modulation in prostate cancer. (A) PPI network composed by TFs identified in enrichment analysis of co‐expressed gene modules and by proteins identified in protein enrichment analysis of genes differentially occupied by transcriptional activation histone marks. Each protein is represented by a node (circle) and their sizes are proportional to their centrality degree. *PVT1* is colored in orange, proteins that interact directly with *PVT1* in LNCaP are colored in red, proteins mostly regulated by AR in pink and regulated by AR‐*PVT1* in purple. Edges represent the direct (solid line) and indirect (dashed line) interactions. (B) Functional enrichment of 38 genes from GO analysis using STRING database. The y‐axis shows the names of enriched pathways; the x‐axis shows the enrichment signal values (weighted harmonic mean between the observed/expected ratio and ‐log[FDR]). Enrichment significance is color scaled from dark blue to light green, representing lower to higher significance, as indicated on the scale at right. Number of genes in the network belonging to the respective pathway is proportional to circle size. In the right panel, GO terms are grouped by terms similarity.

To integrate all our previous findings related to *PVT1* knockdown in LNCaP under androgen treatment, we explored protein–protein interaction network properties using the STRING database.^32^ First, we crossed the results of enrichment analysis of TFs in selected co‐expressed modules (Figure 3D,E) together with enriched PPI gene partners with DO histones (Figure 5C), resulting in 38 proteins. A highly connected network was built with 38 nodes and 147 edges (PPI enrichment analysis, *p* value <1E‐06) (Figure 6A), with an average centrality degree of 7.74, average local clustering of 0.57, and density of 0.2, showing a deeply connected network. Overall, there were nine proteins directly or indirectly interacting with *PVT1* (Figure 6A). Proteins whose genes were mostly affected by AR regulation or AR‐*PVT1* regulation are marked in pink and purple, respectively (Figure 6A). We ranked the proteins by network centrality measures (Figure S5); of note, among the top 10 proteins in the ranking, six had direct (MYC, STAT3, ESR1, and EZH2) or indirect (NFKB1 and TP53) interaction with *PVT1*, suggesting *PVT1* as an important player in this network (Figure 6A).

Functional analysis showed GOs involved with epithelial cell differentiation, proliferation, morphogenesis, and development of prostate gland (Figure 6B). Using KEGG pathways (Table S15), pathways in cancer and prostate cancer were enriched, corroborating that this AR‐*PVT1* network modulation is important in PCa.

## 4 DISCUSSION

Our previous study established that lincRNA *PVT1* interacts with the AR in LNCaP cells and demonstrated that PVT1‐KD13 significantly increased the expression of tumor suppressor genes that were repressed in the presence of this lincRNA.^20^ The present study explores the mechanisms through which *PVT1*, in conjunction with AR, regulates gene expression in PCa, pointing to pro‐oncogenic pathways whose expression is activated by *PVT1*.

*PVT1* transcriptionally activated a set of oncogenic pathways while inhibiting the transcription of tumor suppressor genes. These effects were mediated by *PVT1* acting in a genome‐wide manner through epigenetic mechanisms. Global genome‐wide changes in the occupancy of six different epigenetic marks upon PVT1‐KD13 were mapped by CUT&RUN, and PVT1‐KD13 was found to be associated with significant genome‐wide changes in gene expression. Functional modules enriched in oncogenes were found to be regulated by TFs involved in cancer. *PVT1* affected the deposition of transcription‐activating histone marks in genes associated with pro‐oncogenic functions in response to androgen stimulation. Collectively, these findings resulted in a concise gene network enriched in pathways associated with cancer, suggesting *PVT1* as a critical player in PCa.

Our functional assays on tumor cell proliferation further support the oncogenic role of *PVT1* and demonstrate that our LNCaP model aligns with the existing literature, which documented the oncogenic function of *PVT1* on PCa.^6^ Regarding the effect of *PVT1* on the invasive potential of PCa, it has been shown that *PVT1* silencing inhibits invasion^33^ of DU‐145, an androgen‐insensitive cell line, and of 22Rv1, a cell line that originated from a castration‐resistant tumor, as well as inhibiting proliferation and migration.^33^ Validating our present epigenetic results by testing additional cell lines with different patterns of androgenic regulation and different expression of variant androgen receptors would further strengthen our findings.

To assess the broader clinical relevance of *PVT1*, we analyzed PCa clinical samples exhibiting heterogenous phenotypes. This analysis identified eight distinct gene modules that correlated with *PVT1* expression and were significantly enriched in cancer‐related pathways, including TP53, PI3K/AKT, and mTOR, crucial for cell growth, survival, and metabolism.^34^ In line with this, silencing *PVT1* reduced LNCaP cell proliferation rates compared to CTRL cells.

In the absence of *PVT1*, a notable increase in apoptosis signaling in LNCaP cells was observed. Increased cleaved caspase‐3 levels were significantly more pronounced in PVT1‐KD13 cells compared to CTRL cells, showing that PVT1‐KD13 enhances apoptosis in LNCaP cells. Similar findings were observed in other PCa cell lines such as 22RV1,^33^ DU‐145,^5^, ^33^ and PC‐3^5^ and in other cancer types,^8^, ^35^ in which *PVT1* silencing caused elevated levels of cleaved caspase‐3.

Tumor suppressor gene expression, repressed by androgen, was restored following *PVT1* knockdown in LNCaP cells. Of note, the p53 transcriptional network and the negative regulation of Rho protein signal transduction pathways (Figure 3B) were both identified here as correlated with *PVT1* in PCa clinical samples (Figure 1B). The *p53* gene is mutated or deleted in approximately half of all cancers,^36^ mediating critical processes such as DNA repair, cell cycle arrest, and apoptosis^37^; indeed, TP53 targets are negatively correlated with *PVT1* in PCa clinical samples (Figure 1B). Rho GTPases, a family of small G proteins, are key contributors to tumor initiation and progression with a major role in cell migration and metastasis^27^ and are positively correlated with *PVT1* in PCa clinical samples (Figure 1B).

In the present study, we clearly identified that in the context of AR signaling (i.e., hormone stimulation), *PVT1* increases oncogenes expression. Notably, co‐expression modules M13, M14, M15, and M22 were upregulated by androgen and strongly associated with oncogenic processes driven by *PVT1* in LNCaP (Figure 3D). Genes within these modules (Table S10) were downregulated upon *PVT1* knockdown and are linked to oncogenic processes such as DNA replication, cell cycle, MAPK signaling, and dysregulation of the APC/Rb pathways. MAPK signaling is a well‐characterized pathway implicated in the initiation and progression of PCa.^38^ Adenomatous polyposis coli (APC) and retinoblastoma (RB) tumor suppressor proteins are frequently dysregulated in various cancers, including PCa, and RB loss contributes to tumorigenesis and resistance to androgen deprivation therapy.^39^

Notably, modules M13 and M15 revealed regulation by TFs including E2F4, MAX, and MYC (Figure 3D). In PCa, E2F4 promotes oncogene expression, while its knockdown suppresses malignant features of PCa cells.^40^ MYC forms a heterodimer with MAX to drive transcription of target genes in PCa,^41^ and MYC–*PVT1* interaction in promoting tumorigenesis is widely documented.^10^

The relationship between *MYC* and *PVT1* expression is an important factor to consider, given their proximity in the human genome (with *MYC* located 53 kb upstream of *PVT1*). *PVT1* promoter is contacted by its own four intragenic enhancers, and when *PVT1* promoter is silent, these enhancers engage with *MYC* promoter increasing *MYC* expression.^10^ Here we decreased *PVT1* levels by directly targeting its RNA with CRISPR‐Cas13d without editing the genomic locus promoter, and we observed that *PVT1* knockdown caused a significant 2.1‐fold decrease in *MYC* mRNA expression (false discovery rate [FDR] = 1.09 × 10^23^) (Table S8), showing that an interplay between *PVT1* lincRNA expression levels and *MYC* expression regulation takes place in LNCaP cells, thus warranting further characterization.

Of note, *PVT1* knockdown promoted a global reorganization of chromatin landscape (Table S12); remarkably, a large fraction of genes lost their H3K27me3 marks (2386 genes) and H3K27Ac marks (8943 genes) without corresponding changes in EZH2 occupancy at their loci, showing the epigenetic potential of *PVT1* independently of EZH2 (Figure 4E,F).

A small fraction of genes (226 genes) lost both EZH2 and H3K27ac marks upon *PVT1* KD (Figure 4F and Table S12). This is in line with the finding that EZH2 binds genome‐wide at promoters marked by H3K27ac and acts as a transcription activator, independently of PRC2 and its methyltransferase activity.^42^ This noncanonical function of EZH2 is closely linked to its hidden transactivation domain (TAD), which binds to the transcriptional coactivator and histone acetyltransferase p300, promoting gene expression in a p300‐dependent manner.^43^ Additionally, EZH2 interacts with c‐Myc at non‐PRC2 target sites, using the TAD to recruit coactivators and drive gene activation.^44^ *PVT1* might participate in the recruitment of EZH2 to gene loci that lost H3K27ac marks upon PVT1‐KD13 (Figure 4F and Table S12), a finding that warrants further characterization.

We previously demonstrated that *PVT1*, in cooperation with AR, acts as a transcriptional repressor of hundreds of genes under hormone stimulation in LNCaP cells,^20^ however only the locus of a single gene that was affected by PVT1‐KD13, namely the *NOV* gene, had its histone marks H3K27me3 and H3K27ac analyzed by ChIP‐qPCR.^20^ Here, the use of CUT&RUN permitted the simultaneous genome‐wide mapping in LNCaP cells of six epigenetic marks, which showed that this transcriptional repression induced by *PVT1* involves both AR and H3K27me3 occupancy at thousands of gene loci (Figure 4C,E). Of note, functional enrichment analysis of these differentially affected genes highlighted the Roundabout signaling pathway. This pathway, mediated by Slit proteins and their Roundabout receptors (Slit/Robo), regulates angiogenesis, inflammatory cell chemotaxis, and tumor cell migration and metastasis.^28^ It modulates chemokine‐driven cellular responses and inhibits cell migration by protein degradation via the ubiquitin‐proteasome system in breast cancer.^45^ Slit/Robo signaling exhibits a complex, context‐dependent role in cancer, and knowledge of its role in PCa remains limited, requiring further exploration.

It is well documented that lncRNAs can interact with histone modifiers and thereby regulate gene expression.^46^ For example, in colorectal cancer *PVT1* recruits SF1 to the *FDX1* promoter, favoring H3K27ac deposition and increasing *FDX1* transcription.^47^ Our findings strongly indicate that *PVT1* cooperates with AR to facilitate histone modifications associated with transcriptional activation—specifically H3K4me1, H3K4me3, and H3K27ac—at oncogenic gene *loci* involved in Rho GTPase/RAC1 signaling, MAPK signaling, the VEGFA‐VEGFR2 pathway, and the Hippo signaling pathway, all of which play notable oncogenic functions. Regarding the Hippo pathway, several main players had their expression reduced in LNCaP after PVT1‐KD13 under androgen treatment (*WWTR1[TAZ], WWC1, MOB1B* and *AMOT*), while *SAV1, WWC3* and *YAP1* showed an increase in expression (Figure S6). Modulation of gene expression was accompanied by a decrease in deposition of transcriptional activation histone marks after PVT1‐KD13 under androgen treatment (Figure S6), suggesting that most members of Hippo signaling are upregulated in the presence of *PVT1*, including *TAZ* (*WWTR1*), which is considered an oncogene. Under hormone treatment several PCa stem cell markers were modulated, of which five were upregulated (*ITGA6, CXCR4, TGM2, EZH2, TACSTD2*) (Figure S7), being mainly involved with chemoresistance and invasion. Their expression was decreased after PVT1‐KD13 (Figure S7), suggesting an increase in the stemness state of the cells in the presence of *PVT1*. Interestingly, after PVT1‐KD13 under hormone treatment, two stem cell markers had their expression increased compared to cells only treated with hormone, *ALCAM (CD166)* and *PSCA* (Figure S7); both are increased in Castration‐Resistant Prostate Cancer (CRPC).^48^, ^49^ The precise molecular interactions and potential co‐factors involved in the *PVT1*–AR axis in PCa, such as for example *PVT1* direct interaction with acetyltransferases or demethylases, remain to be fully characterized.

Enrichment analysis of PPI partners (Table S14) of genes whose proteins showed a significant decrease in transcriptionally activating histone marks following PVT1‐KD13 pointed to possible specific molecular mechanisms; a decrease in the enhancer mark H3K4me1 occurred at PPI partners of a set of proteins that included signaling molecules ESR1, AR, TP53, GSK3B, CTNNB1, MAPK14, RB1, CDK2, and STAT3 (Figure 5C and Table S14). In contrast, proteins whose genes had a decrease in promoter‐associated transcriptionally activating marks (H3K4me3, H3K27ac) following PVT1‐KD13 were PPI partners of proteins related to broader functional categories, including cell cycle regulators, nuclear hormone receptors, key mediators of cellular processes, and TFs (Figure 5C and Table S14). The PPI network of proteins whose genes had decreased H3K4me3 and H3K27ac occupancy following PVT1‐KD13, identified here, may act as co‐regulators—either as co‐activators or co‐repressors—of TFs such as MYC, STAT3, ATF2, NFKB1, NFKB2, and RELA (Figure 5C); a comprehensive list of these partners is provided in Table S14, offering multiple avenues for further investigation. Noteworthy, STAT3 appeared as a top ranked TF that was modulated by PVT1‐KD13 in LNCaP (Figures 6 and S5), and STAT3 known PPI network partners exhibited a significant loss in transcriptionally activating histone marks and a significant decrease in expression upon PVT1‐KD13 (Figure 5C and Table S14), suggesting that *PVT1* is an activator of the STAT3 pathway in prostate cancer, thus warranting further investigation. Interestingly, *PVT1* lncRNA binds to phospho‐STAT3^50^ and activates the STAT3 pathway in gastric cancer,^50^ in hepatoblastoma^51^ and in osteosarcoma.^52^

In conclusion, our groundwork points to a gene network modulated by *PVT1*‐AR, which is enriched in PCa genes, providing the basis for further exploring the specific mechanisms by which *PVT1* influences the deposition of transcriptionally activating and repressive histone marks and modulates both oncogenes and tumor suppressor genes through a complex modulatory interplay.

## AUTHOR CONTRIBUTIONS

**Maria Gabriela Berzoti‐Coelho:** Conceptualization; investigation; formal analysis; writing – original draft; writing – review and editing; methodology; data curation; visualization. **Fabio Nunes de Mello:** Methodology; software; data curation; investigation; formal analysis; writing – original draft; writing – review and editing; visualization. **Ana Carolina Tahira:** Software; formal analysis; data curation; writing – original draft; writing – review and editing; visualization. **Gabriel Nakanishi Fortes:** Methodology; investigation; validation. **Agatha Fischer‐Carvalho:** Methodology; investigation; validation. **Pedro Jardim Poli:** Methodology; investigation; validation. **Murilo Sena Amaral:** Writing – review and editing; funding acquisition; supervision. **Sergio Verjovski‐Almeida:** Conceptualization; formal analysis; funding acquisition; project administration; writing – original draft; writing – review and editing; supervision.

## FUNDING INFORMATION

The study was supported by São Paulo Research Foundation [grant number 2018/23693‐5 to Sergio Verjovski‐Almeida and fellowships 2020/02976‐9 to Maria Gabriela Berzoti‐Coelho, 2022/11192‐7 to Fabio Nunes de Mello, 2024/16545‐0 to Agatha Fischer‐Carvalho, 2024/16644‐9 to Pedro Jardim Poli and 2021/13698‐2 to Gabriel Nakanishi Fortes] and by Fundação Butantan. Sergio Verjovski‐Almeida received a Research Career Fellowship award from Conselho Nacional de Desenvolvimento Científico e Tecnológico (CNPq 313781/2025‐7). The funders had no role in the study design, data collection, analysis, the decision to publish, or the preparation of the manuscript.

## CONFLICT OF INTEREST STATEMENT

The authors declare that they have no competing interests.

## Supporting information


**DATA S1.** Supporting Information.

**TABLE S5.** Coverage and quality metrics of sequencing data for CUT&RUN and RNA seq.

**TABLE S6.** WGCNA co‐expression modules for the genes expressed in the PCa samples of the TCGA database.

**TABLE S7.** Gene functional enrichment analysis of gene co‐expression modules in the TCGA PCa samples database.

**TABLE S8.** Differentially expressed genes in RNA‐seq analyses of LNCaP cells treated with hormone.

**TABLE S9.** Functional enrichment analysis of genes differentially expressed in the RNA seq analyses of LNCaP cells treated with hormone or under *PVT1* knockdown.

**TABLE S10.** Genes in WGCNA co‐expression modules for RNA‐seq data from LNCaP cells.

**TABLE S11.** Peaks from CUT&RUN experiments regarding AR, EZH2, H3K4me3, H3K27ac, H3K27me3 and H3K4me1 genome‐wide occupancy.

**TABLE S12.** Genes differentially occupied by EZH2, H3K27me3, H3K27ac, AR, H3K4me3, and H3K4me1.

**TABLE S13.** Functional enrichment analysis of genes differentially occupied by AR and H3K27me3.

**TABLE S14.** Enrichment analysis of GOs or of known Protein–Protein Interaction (PPI) partners differentially occupied by transcriptionally activating histone marks or differentially expressed upon *PVT1 KD*.

**TABLE S15.** Functional enrichment pathways analysis of Protein–Protein Interaction (PPI) network.

## Data Availability

The raw data of CUT&RUN and RNA‐seq is available at GenBank SRA under the Bioproject accession number PRNJA1313626. Scripts used to analyze the dataset are available at github page (https://github.com/actahira/PVT1-lincRNA-signals). Genome tracks for the CUT&RUN data, in the human UCSC browser hg38 format, are available at zenodo https://doi.org/10.5281/zenodo.17137240. Further information is available from the corresponding author upon reasonable request.
