# Supplementary material for: PVT1 lincRNA signals an androgen‐dependent transcriptional activation program of oncogenes in prostate cancer cells
Source: Int J Cancer. 2026 Mar 6;159(1):144–58. doi: 10.1002/ijc.70417 (PMC13139993; doi:10.1002/ijc.70417)
Supplement: Supplementary file 1 — DATA S1. Supporting Information. [file IJC-159-144-s008.pdf]

Supplementary Material

PVT1 lincRNA signals an androgen-dependent transcriptional activation program of oncogenes in prostate cancer cells

Maria Gabriela Berzoti-Coelho, Fabio Nunes de Mello, Ana Carolina Tahira, Gabriel Nakanishi Fortes, Agatha Fischer-Carvalho, Pedro Jardim Poli, Murilo Sena Amaral, Sergio Verjovski-Almeida

Table of Contents

Supplementary Methods 2

Supplementary Tables 9

Tables S1 to S4 9

Tables S5 to S15 – available in separate excel files 10

Supplementary Figures S1 to S7 11

Supplementary References 16

Supplementary Methods

Cell culture

Cells were cultured in RPMI medium (Gibco, USA) supplemented with 10% fetal bovine serum, 4.5 g/L glucose, 1.5 g/L sodium bicarbonate, 2.4 g/L HEPES, 1% sodium pyruvate, and 1% penicillin (100 U/mL)/streptomycin (100 U/mL) solution. Cells were grown to 70–100% confluency. Subculturing was performed two to three times/week using trypsin-EDTA (Gibco), with a split ratio of 1:2 to 1:5. Cells were maintained at 37 °C in a humidified atmosphere containing 5% CO_2.

Cloning

gRNA sequences (**Table S1**, below) were cloned into the pLentiRNAGuide vector (Addgene #138151). Oligonucleotides were first hybridized and phosphorylated using Anza PNK (Invitrogen) under the following conditions: 20°C for 15 min, 80°C for 5 min, 95°C for 1 min, followed by a gradual temperature ramp down to 25°C.

The hybridized and phosphorylated oligos were then inserted into the BsmBI cloning site using Golden Gate assembly. Reactions included T4 DNA Ligase Master Mix (Invitrogen) and Esp3I (Invitrogen) and were carried out for 15 cycles of 37°C for 5 min and 20°C for 5 min, followed by 80°C for 5 min, and held at 20°C indefinitely.

The resulting pLentiRNAGuide constructs — PVT1-KD13, PVT1-KD17, and CTRL — were used in combination with pLentiRNACRISPR (Addgene #138149) for lentiviral production.

Bacterial Transformation

Ligation products were transformed into HB101 (Promega) or Top10 (Invitrogen) chemically competent cells. Resulting colonies were screened by colony PCR and confirmed by Sanger sequencing.

Lentiviral Production

Lentiviral production was performed in HEK293T cells (ATCC Cat# CRL-3216), cells were seeded at 3×10^6 cells per 10 cm dish 48 h prior to transfection and maintained at 37°C with 5% CO_2. Cells were transfected with a lentiviral packaging mix containing 15 µg of the lentiviral construct (cloned pLentiRNAGuide or pLentiRNACRISPR), 10 µg of packaging plasmid (psPAX2, Addgene #12260), 5 µg of envelope plasmid (pMD2.G, Addgene #12259), and Lipofectamine 2000 (Invitrogen), following the manufacturer's protocol. Lentiviral supernatants were harvested 48 h post-transfection, filtered through 0.45 µm filters (Millipore Cat# SLHV033RS), and stored at –80°C.

Viral titration

Lentiviral titers were determined in T98G cells (ATCC Cat# CRL-1690) using Addgene's Colony Formation Titering Assay protocol [1].

Viral Transduction

One million LNCaP cells were seeded 48 h prior to transduction. Cells were transduced with lentiviral supernatants at an MOI of 0.006 for pLentiRNACRISPR or 15 for pLentiRNAGuide. After 48 h, cells transduced with pLentiRNACRISPR were selected with 15 µg/mL Blasticidin (Gibco Cat# A1113903). Subsequently, these cells were transduced with pLentiRNAGuide viral supernatants (PVT1-KD13, PVT1-KD17 or CTRL) for 48 h at an MOI of 15 and then selected with 1 µg/mL Puromycin (Gibco Cat# A1113803). Both Blasticidin and Puromycin selections were maintained with medium replacement every 48 h until pure populations of transduced cells were established.

To induce *PVT1* knockdown, LNCaP transduced cells were treated with 2 µg/mL doxycycline hydrochloride (Sigma-Aldrich) for 96 h, with the medium refreshed and doxycycline replaced at 48 h of culture.

RNA extraction, cDNA synthesis, and RT-qPCR

The cells were centrifuged at 340 × g for 4 min at 4°C, and the resulting pellet was washed with 1x PBS. Following a second centrifugation, the pellet was resuspended in 700 µL of TRIzol reagent (Invitrogen). Next, 140 µL of chloroform was added, the mixture was vortexed for 15 s and incubated at room temperature for 2 min. The samples were then centrifuged at 12,000 × g for 15 min at 4°C. The aqueous phase was carefully transferred to a microtube containing an equal volume of 70% ethanol and subsequently loaded onto micro-spin columns from the RNeasy Micro Kit (Qiagen, USA). The remaining steps of RNA extraction were performed following the manufacturer's protocol.

For cDNA synthesis, 500 ng to 1 µg of total RNA was used in reverse transcription (RT) reactions with the SuperScript IV First-Strand Synthesis System (Invitrogen) and random hexamer primers, following the manufacturer's instructions. Real-time quantitative PCR (qPCR) was performed using gene-specific primers for the target genes (**Table S2**). GAPDH (Glyceraldehyde-3-Phosphate Dehydrogenase) and ACTB (Beta-Actin) served as endogenous controls. qPCR reactions were prepared using 1/32 of the cDNA synthesis reaction, SYBR Green I Master Mix (Roche), and primers at a final concentration of 10 µM. Amplification was carried out on a LightCycler 480 II system (Roche), with three technical replicates per sample. Gene expression levels were normalized to the average expression of the endogenous controls and presented as fold change.

Proliferation Assays

Trypan blue exclusion assays were conducted using 3.5 × 10^5 LNCaP cells/well (CTRL or PVT1-KD13 / PVT1-KD17) seeded in 6-well plates. After 48h of incubation to ensure complete cell adhesion (designated as day zero), cell proliferation was evaluated for 5 days at 24h intervals. Each day, one well from each condition was trypsinized, and cells were stained with 0.4% Trypan Blue Stain (Invitrogen) at a 1:1 ratio, followed by automated cell counting (Countess II FL, Thermo Fisher Scientific, USA).

To ensure accuracy, six different cell-to-trypan blue dilutions (all at a 1:1 ratio) were prepared for each condition. Cell counts were normalized to the respective values obtained on day zero. The non-parametric Mann-Whitney test was used, with p-values <0.05 considered statistically significant.

Invasion Assays

Fluoroblok transwell inserts (Becton Dickinson, USA) with 8 µm pores were pre-coated with Geltrex (Gibco) diluted 1:50 in coating buffer (0.01 M Tris-HCl, pH 8, 0.7% NaCl). Each experimental condition was performed in technical duplicates.

CTRL or PVT1-KD13 / PVT1-KD17LNCaP cells (1.7 × 10^5 cells) were washed with PBS, resuspended in 300 µL of serum-free medium, added to the Geltrex-coated inserts, and then placed in a 24-well support plate (Corning, USA). In the lower chamber (beneath the insert), 700 µL of medium supplemented with 20% FBS was added as a chemoattractant. Cells were incubated for 48h to allow for invasion.

At the end of the incubation period described in the main text, cells were stained with 1 µg/mL calcein, and those that had migrated to the underside of the membrane were visualized using a fluorescence microscope. To ensure complete coverage, 18 distinct fields were imaged per insert.

For each condition, input control wells (technical triplicates) were included by seeding the same number of cells used in the inserts. After the invasion assay, these wells were fixed with 100% methanol and stained with 0.1% crystal violet in a solution of 10% methanol and 90% water. Following washing and drying, the dye was eluted with 15% acetic acid, and absorbance was measured at 595 nm using a plate reader.

Fluorescence images were analyzed using ImageJ software for automated quantification of invaded cells. Cell counts were normalized by dividing the number of invaded cells by the average absorbance value of the corresponding input condition.

Statistical analysis was conducted using the non-parametric Mann-Whitney test, with p-values < 0.05 considered statistically significant.

Apoptosis Assays

Protein concentrations from the lysates were determined using the Pierce BCA Protein Assay Kit (Thermo Fisher Scientific, USA), according to the manufacturer's protocol. For protein expression analysis by western blot, 50 µg of total protein was loaded onto a 15% acrylamide gel and separated by SDS-PAGE.

After electrophoresis, proteins were transferred to a PVDF membrane using a semi-dry transfer system. The membrane was washed once with TBS-T and then blocked for 1 h at room temperature in 5% BSA diluted in TBS-T. Primary antibody incubation was performed overnight at 4°C using the following antibodies: anti-tubulin (loading control, 1:1,000; Cell Signaling Technology), anti-caspase-3 (1:500; Cell Signaling Technology), and anti-cleaved caspase-3 (1:500; Cell Signaling Technology).

After incubation, membranes were washed three times for 10 min each with TBS-T and then incubated with an anti-rabbit secondary antibody (Merck-Millipore) diluted 1:10,000 for 1 h at room temperature.

CUT&RUN

At the end of the CUT&RUN or sonication reactions, the resulting DNA was extracted using the DNA Purification Buffers and Spin Columns Kit - ChIP, CUT&RUN (Cell Signaling Technology #14209), following the manufacturer's instructions.

The purified DNA was subsequently used for sequencing library preparation using the SimpleChIP ChIP-seq DNA Library Prep Kit for Illumina (Cell Signaling Technology #56795) and the SimpleChIP ChIP-seq Multiplex Oligos for Illumina (Dual Index Primers) (Cell Signaling Technology #47538), following the manufacturer's guidelines. DNA concentration was quantified using a Qubit fluorometer (Thermo Fisher Scientific), and sample quality was evaluated with the High Sensitivity RNA Kit (Agilent) on a Bioanalyzer system (Agilent). The prepared DNA libraries were multiplexed and sequenced on a DNBseq (BGI Genomics, China), with 50bp paired-end sequencing for CUT&RUN samples and 100bp paired-end sequencing for input samples.

CUT&RUN data analysis

Sequencing reads were trimmed and cleaned with fastp v.020.0 [2]. Alignment for CUT&RUN reads was done with bowtie2 v.2.3.5.1 [3] with standard --very-sensitive-local parameters, against the GENCODE 40 [4] reference. CUT&RUN peaks were called with MACS3 v.3.0.0a6 [5], with parameters --nolambda -q 0.2, and consensus peaks were generated per antibody by grouping peaks from the individual samples, using the input samples as background. Peak calling quality was assessed with PeakQC v.0.1.3 [6] and

deeptools v.3.3.2 [7]. Peaks were annotated to the nearest gene with ChIPseeker [8] against the GENCODE 40 annotation, considering promoter regions up to 5kb upstream of transcription start. Peaks were merged across all samples of the same antibody, and quantified with samtools v.1.10 [9]; counts were normalized against Greenlist counts [10] as previously described by our group. Briefly, our pipeline uses the Shannon's Entropy to find the genomic regions that are well represented and distributed in all our samples. Regions with low entropy have stochastic representation in samples, while regions with high entropy have proportional representation in almost all our samples, being more homogeneously distributed in our dataset. These regions are represented by bins and usually are well correlated with library size, accounting for technical artifacts variation and have a high correlation among the samples in the dataset. We chose 0.1% of the highest correlated regions, named as Greenlist. To calculate the normalization factor, we calculated sum of Greenlist counts for each sample and divided each sum by the median sum across all samples. This outperformed other methods in the literature (see Greenlist article)[10]. The peaks delineated by MACS3 analysis and normalized by Greenlist were subjected to statistical tests performed with DESeq2 [11], comparing R1881 androgen versus CTRL, PVT1-KD13 vs CTRL without or with R1881 androgen treatment for each epigenetic mark. Two biological replicates per each epigenetic mark sample were assayed. Only those regions that presented a differential occupancy at an adjusted p-value ≤ 0.05 were considered significant. CUT&RUN sequencing coverage and sequence quality statistics of each sample are described in **Table S5**.

RNA-seq data analysis

Sequencing reads were trimmed and cleaned with fastp v.020.0 [2]. RNA reads were aligned with STAR v.2.7.3a [12], with default parameters, against the the GENCODE 40 [4] reference; transcript quantification was measured with featureCounts [13], and statistical tests were performed with edgeR [14] and sva [15]. RNA-seq sequencing coverage and sequence quality statistics of each sample are described in **Table S5**.

Enrichment analyses

Enrichment analyses of gene sets were done through Enrichr [16], with annotations for Reactome [17], WikiPathways [18] and NCATS BioPlanet [19] databases, GO terms, ChEA [20] and protein-protein interaction annotation. Significance was defined as adjusted p≤0.05, and significant terms/pathways of interest were selected for discussion; pathways were selected based on lowest p-value or, in case of figures with multiple datasets (such as PPI enrichment results, Figure 5c), terms were selected by the lowest average rank after ranking the p-values of individual datasets.

Co-expressed network modules and module preservation

Gene co-expression analyses were done with weighted gene co-expression network analysis (WGCNA) [21], with a soft thresholding power of 12 and a signed network. Module splitting parameters were changed to reassignThreshold = 0, mergeCutHeight = 0.1, deepSplit = 2, in order to generate smaller, more nuanced modules. To increase the statistical power, the LNCaP untreated RNA-seq samples were included for calculating co-expression.

Module preservation between RNA-seq and CUT&RUN antibody sample datasets for each gene co-expression module was assessed with the WGCNA function *modulePreservation*, using biweight correlation and signed network. For CUT&RUN data, occupancy by an epigenetic mark within 2 kb of the promoter region of each gene in the module was quantified in each antibody sample, normalized by Greenlist, and normalized control IgG counts at the promoter of each gene were subtracted for each sample to minimize genomic noise. The goal of the analysis was to assess whether RNA co-expression modules also showed correlated deposition of each epigenetic mark assayed, i.e. whether the mark deposition of these genes also show module-like features, which could indicate the action of a common epigenetic regulator. For each co-expression module, seven preservation metrics (propVarExplained, meanSignAwareKME, meanSignAwareCorDat, meanAdj, cor.kIM, cor.kME, and cor.cor), as defined by Ritchie et al. [22] were evaluated against 10,000 randomized permutations, randomizing module assignments across genes. A significantly preserved metric was defined as a performance superior to 95% of permutations for each CUT&RUN antibody sample but inferior to 5% of permutations in control IgG samples (i.e. preservation metrics higher than random for a given epigenetic mark, but lower than random for the control samples, to control for false positives created by genomic background counts). Modules with at least 6 out of 7 significantly preserved metrics, as recommended by Ritchie et al. [22], were considered as preserved between RNA-seq and CUT&RUN antibody sample datasets.

For TCGA analysis, all 528 prostate cancer samples from 497 patients with available RNA-seq data in the TCGA-PRAD cohort (https://portal.gdc.cancer.gov) were selected, normalized as counts per million, and genes with a coefficient of variation less than 0.5 were discarded. Co-expression module identification was done with WGCNA, using signed networks and a soft thresholding power of 4; module-eigengene correlations were Z-score normalized by module for plotting. Module correlation to *PVT1* expression was assessed with a generalized linear model.

PPI network analysis with STRING

PPI network analysis was performed using STRING database [23]; only physical interactions database was used to reduce weaker interaction, and redundant interactions were filtered. Genes from TFs enrichment analysis of co-expressed modules from WGCNA [21] analysis were selected. Modules used were those affected by androgen treatment after *PVT1 KD* (M04-06, M12-15, M22 and M24) and those affected by androgen treatment (M03, M07, M08, M17-21 and M23). We also used genes from PPI enrichment analysis from genes differentially occupied by gene activation marks (H3K4me1, H3K4me3 and H3K27Ac). Together, these datasets comprised a set of 38 genes that were used as input in PPI network analysis. Edges were from STRING database physical interactions. Node centrality measures were calculated using igraph [24] from the R platform [25].

To search for lncRNA interaction with protein, we used the NPInter v5.0 [26] database, which brings all types of interactions of lncRNAs, and used only RNA-protein interactions. We also used the LncTarD database [27], a database of functional lncRNA-target regulations in human diseases supported by experimental analysis.

Supplementary Tables

Tables S1 to S4

Table S1: gRNA sequences targeting *PVT1*

Oligonucleotide		Sequence 5' - 3'
PVT1-KD13	sense	CGGATGGAACGTGCATCAGATGC
	antisense	GCATCTGATGCACGTTCCATCCG
PVT1-KD17	sense	GCAGTCCTTCGTCCCCCATGGAC
	antisense	GTCCATGGGGGACGAAGGACTGC

Table S2: RT-qPCR primers sequence

Primer		Sequence 5' – 3'
ACTB	Forward	CTTCCTTCCTGGGCATGG
	Reverse	AGACAGCACTGTGTTGGCGTA
GAPDH	Forward	CTCTCTGCTCCTCCTGTTCGA
	Reverse	ACGACCAAATCCGTTGACTCC
PVT1	Forward	TGGAATGTAAGACCCCGACTCT
	Reverse	GATGGCTGTATGTGCCAAGGT

Table S3: Antibodies for CUT&RUN Assays

Antibody	Manufacturer	Reference code #	Volume Applied for 100,000 cells*
H3K4me3 antibody ChIP-seq Grade	Cell Signaling Technology	C42D8	2 μL
H3K27ac antibody ChIP-seq Grade	Diagenode	C15410196	4,6 μL from 1:10 dilution
H3K27me3 antibody ChIP-seq Grade	Diagenode	C15410195	11,8 μL from 1:10 dilution
H3K4me1 antibody ChIP-seq Grade	Diagenode	C15410194	8,7 μL from 1:10 dilution
Anti-Androgen Receptor Antibody	MilliporeSigma	06-680	4,3μL
Ezh2 (D2C9) XP® Rabbit mAb	Cell Signaling Technology	5246S	2,9 μL
Rabbit mAb IgG-XP® Isotype Control	Cell Signaling Technology	66362	5 μL

*Volumes calculated according to the antibody mass indicated for Cut&Run reactions in each antibody's datasheet

Table S4: Sonication parameters at Covaris® S2

Sample	Duty Cycle (%)	Intensity	Cycles per Burst	Time (s)
**CTRL**	2	6	200	840
**PVT1-KD13**	2	6	200	480

Tables S5 to S15 – available in separate excel files

Table S5: Coverage and quality metrics of sequencing data for CUT&RUN and RNA-seq

Table S6: WGCNA co-expression modules for the genes expressed in the PCa samples of the TCGA database

Table S7: Gene functional enrichment analysis of gene co-expression modules in the TCGA PCa samples database

Table S8: Differentially expressed genes in RNA-seq analyses of LNCaP cells treated with hormone

Table S9: Functional enrichment analysis of genes differentially expressed in the RNA-seq analyses of LNCaP cells treated with hormone or under *PVT1* knockdown

Table S10: Genes in WGCNA co-expression modules for RNA-seq data from LNCaP cells

Table S11: Peaks from CUT&RUN experiments regarding AR, EZH2, H3K4me3, H3K27ac, H3K27me3 and H3K4me1 genome-wide occupancy

Table S12: Genes differentially occupied by EZH2, H3K27me3, H3K27ac, AR, H3K4me3, and H3K4me1

Table S13: Functional enrichment analysis of genes differentially occupied by AR and H3K27me3

Table S14: Enrichment analysis of GOs or of known Protein-Protein-Interaction (PPI) partners differentially occupied by transcriptionally activating histone marks or differentially expressed upon *PVT1* KD.

Table S15: Functional enrichment pathways analysis of Protein-Protein-Interaction (PPI) network.

Supplementary Figures S1 to S7

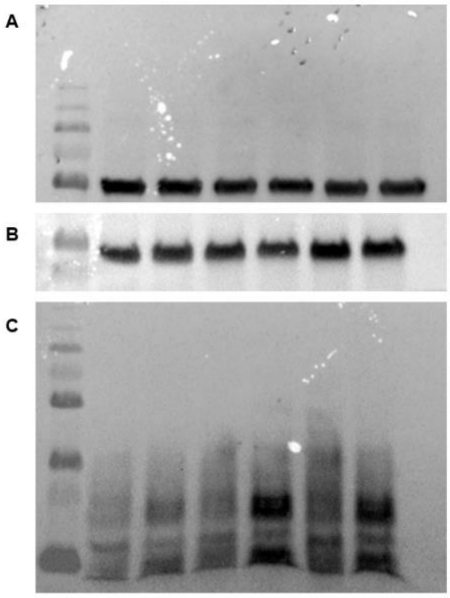

Figure S1: ***LNCaP Casp3 WB Membranes.*** Protein expression and molecular weight markers of LNCaP CTRL and PVT1-KD13 / PVT1-KD17 cells under staurosporine treatment: (**A**) Tubulin (55 kDa); (**B**) Pro-caspase 3 (35 kDa); (**C**) Cleaved caspase 3 (19 kDa and 17 kDa).

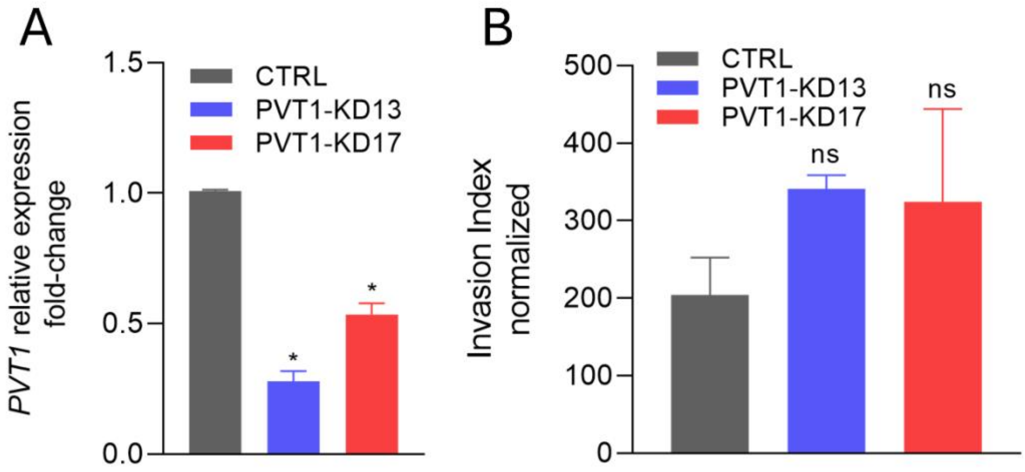

Figure S2: *LNCaP Invasion Assay.* Invasion of LNCaP cells: (**A**) *PVT1* levels measured with RT-qPCR in an aliquot of CTRL, PVT1-KD13, or PVT1-KD17 cells that were used in invasion assays; (**B**) invasion levels of CTRL, PVT1-KD13, and PVT1-KD17 after 48h of invasion. Statistical analysis performed with Mann-Whitney test. Graphs show the mean (+/- S.D.) of three biological replicates. *p < 0.05

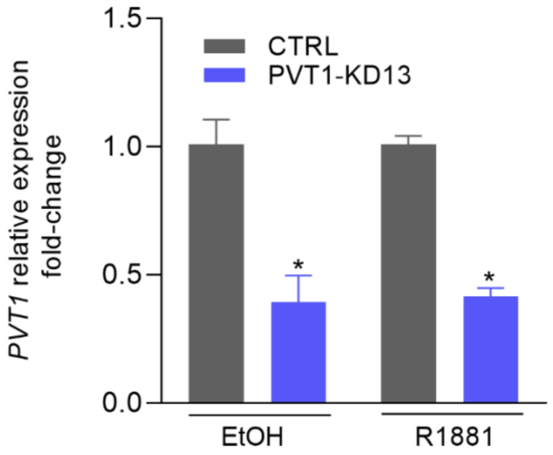

Figure S3: *PVT1* knockdown levels in RNA-seq assays: expression of *PVT1* measured with RT-qPCR in aliquots from the three biological replicates of CTRL or PVT1-KD13 cells that were used in RNA-seq assays, following treatment with R1881 or with the corresponding volume of vehicle (EtOH).

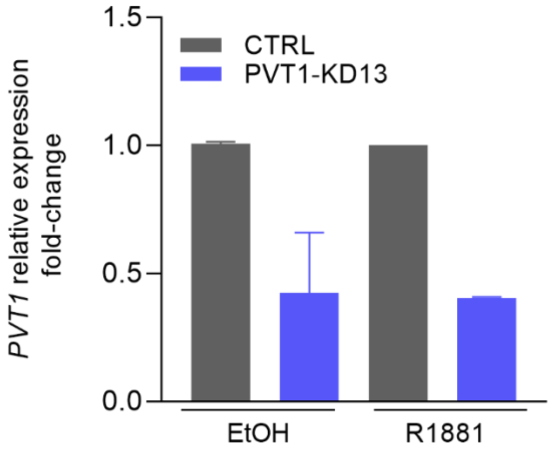

Figure S4: *PVT1* knockdown levels in CUT&RUN assays: expression of *PVT1* measured with RT-qPCR in aliquots from the two biological replicates of CTRL or PVT1-KD13 cells that were used in CUT&RUN assays, following treatment with R1881 or with the corresponding volume of vehicle ethanol (EtOH).

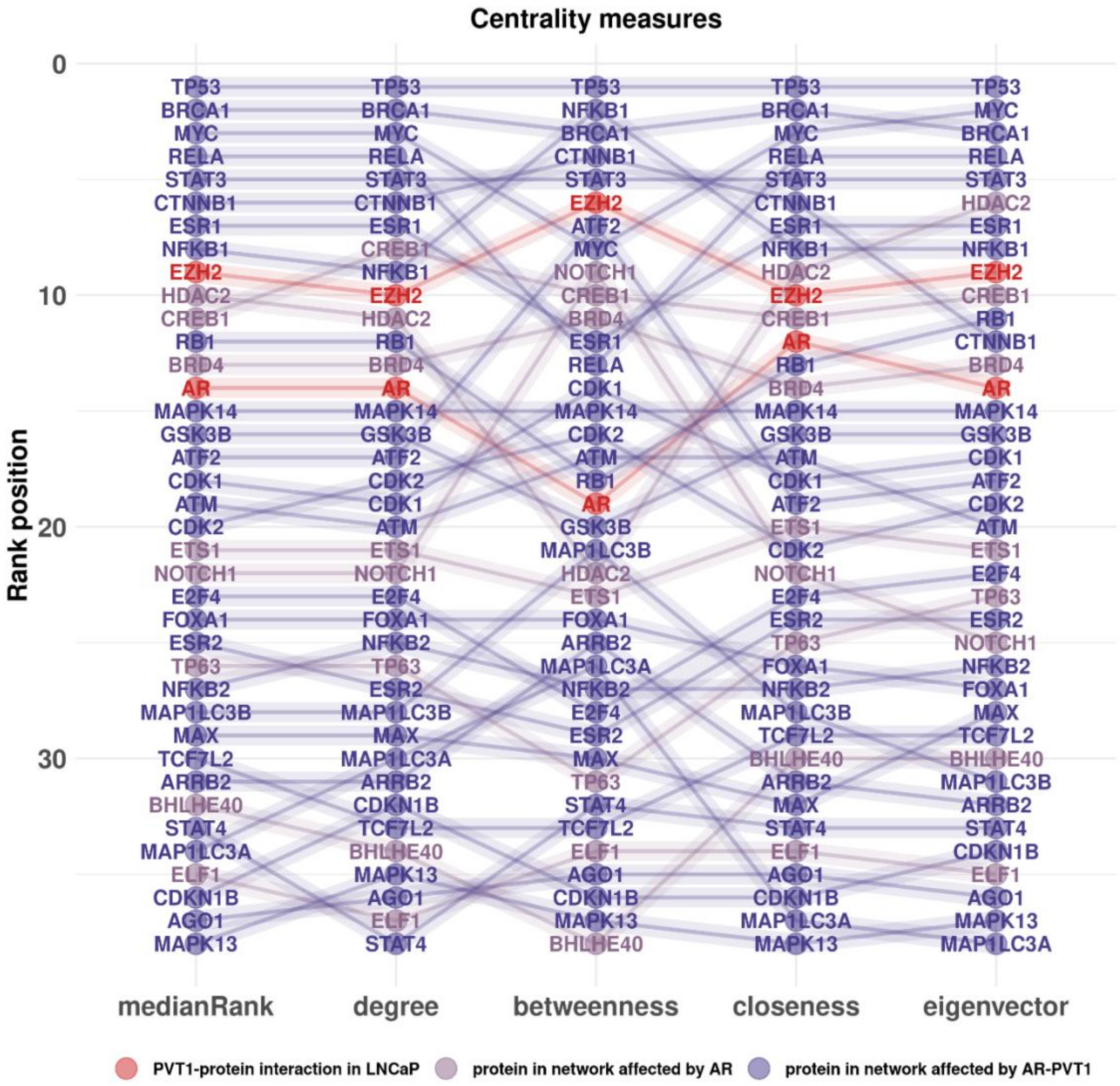

Figure S5: Node centrality measures: In the x axis, centrality measures are represented (degree, betweenness, closeness and eigenvector) together with the median rank of these measures. In the y axis, the ranking position of each protein is represented. Proteins are represented by circles and respectively named. Proteins that interact directly with *PVT1* in LNCaP are colored in red, proteins mostly regulated by AR in pink and regulated by AR-*PVT1* in purple.

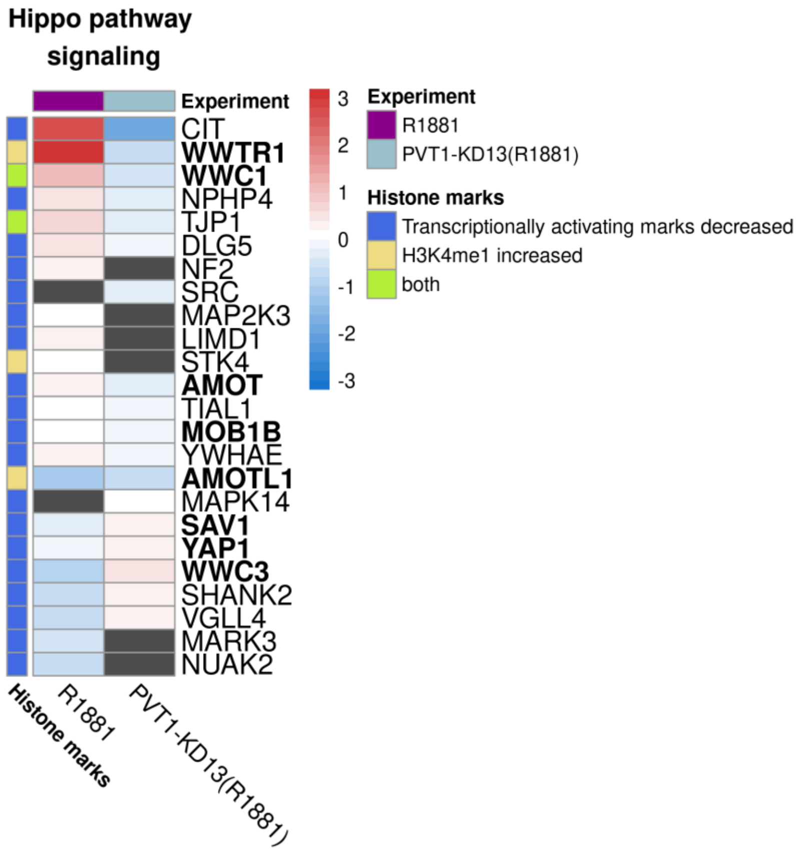

Figure S6: **Heatmap of gene expression levels of members of the Hippo pathways enriched in the histone marks analysis.** The member genes are shown in the lines, and columns show the RNA-seq differential gene expression analysis, color-scaled by log_2FC from blue ($log_2FC < 0$) to red ($log_2FC > 0$). Each experiment is indicated by a different color label at the top of the column: *dark magenta*, R1881 represents the results from RNA-seq analysis of LNCaP cells in the presence of R1881 hormone versus Control (no hormone); *light blue*, PVT1-KD13(R1881) is the analysis comparing LNCaP PVT1-KD13 cells in the presence of R1881versus R1881 treated cells (no knockdown). The different colors in the column at the left side of the heatmap, labeled “Histone marks”, indicate: *blue*, genes with decreased deposition of transcriptionally activating histone marks after *PVT1* knockdown under hormone treatment; *yellow*, genes with increased H3K4me1 deposition upon hormone addition; and *green*, genes with both characteristics. Gene names in bold are those with main roles in Hippo pathways.

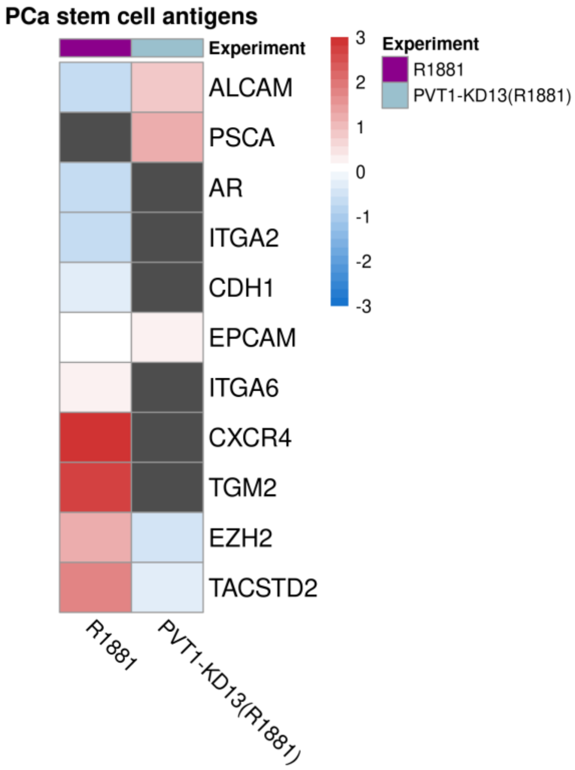

Figure S7: Heatmap of gene expression of members of PCa stem cell antigens. The stem cell antigen genes are shown in the lines, and columns show the RNA-seq differential gene expression analysis, color-scaled by log_2FC from blue ($log_2FC < 0$) to red ($log_2FC > 0$). Each experiment is indicated by a different color label at the top of the column: *dark magenta*, R1881 represents the results from RNA-seq analysis of LNCaP cells in the presence of R1881 hormone versus Control (no hormone); *light blue*, PVT1-KD13(R1881) is the analysis comparing LNCaP PVT1-KD13 cells in the presence of R1881 versus R1881 treated cells (no knockdown).

Supplementary References

1. Addgene. Colony Titering Assay for Lentiviruses [Internet]. 2021 [cited 2021 Apr 9];Available from: https://www.addgene.org/protocols/colony-formation-titering-assay/

2. Chen S, Zhou Y, Chen Y, Gu J. fastp: an ultra-fast all-in-one FASTQ preprocessor. *Bioinformatics* 2018;34:i884–90.

3. Langmead B. Aligning Short Sequencing Reads with Bowtie [Internet]. In: Current Protocols in Bioinformatics. Hoboken, NJ, USA: John Wiley & Sons, Inc., 2010. Available from: http://doi.wiley.com/10.1002/0471250953.bi1107s32

4. Frankish A, Diekhans M, Jungreis I, Lagarde J, Loveland JE, Mudge JM, Sisu C, Wright JC, Armstrong J, Barnes I, Berry A, Bignell A, et al. GENCODE 2021. *Nucleic Acids Res* 2021;49:D916–23.

5. Zhang Y, Liu T, Meyer CA, Eeckhoute J, Johnson DS, Bernstein BE, Nussbaum C, Myers RM, Brown M, Li W, Liu XS. Model-based Analysis of ChIP-Seq (MACS). *Genome Biol* 2008;9:R137.

6. Carroll TS, Liang Z, Salama R, Stark R, De Santiago I. Impact of artifact removal on ChIP quality metrics in ChIP-seq and ChIP-exo data. *Front Genet [Internet]* 2014 [cited 2025 Aug 27];5. Available from: http://journal.frontiersin.org/article/10.3389/fgene.2014.00075/abstract

7. Ramírez F, Dündar F, Diehl S, Grüning BA, Manke T. deepTools: a flexible platform for exploring deep-sequencing data. *Nucleic Acids Res* 2014;42:W187–91.

8. Yu G, Wang L-G, He Q-Y. ChIPseeker: an R/Bioconductor package for ChIP peak annotation, comparison and visualization. *Bioinformatics* 2015;31:2382–3.

9. Danecek P, Bonfield JK, Liddle J, Marshall J, Ohan V, Pollard MO, Whitwham A, Keane T, McCarthy SA, Davies RM, Li H. Twelve years of SAMtools and BCFtools. *GigaScience* 2021;10:giab008.

10. De Mello FN, Tahira AC, Berzoti-Coelho MG, Verjovski-Almeida S. The CUT&RUN greenlist: genomic regions of consistent noise are effective normalizing factors for quantitative epigenome mapping. *Brief Bioinform* 2024;25:bbad538.

11. Love MI, Huber W, Anders S. Moderated estimation of fold change and dispersion for RNA-seq data with DESeq2. *Genome Biol* 2014;15:550.

12. Dobin A, Davis CA, Schlesinger F, Drenkow J, Zaleski C, Jha S, Batut P, Chaisson M, Gingeras TR. STAR: ultrafast universal RNA-seq aligner. *Bioinformatics* 2013;29:15–21.

13. Liao Y, Smyth GK, Shi W. featureCounts: an efficient general purpose program for assigning sequence reads to genomic features. *Bioinformatics* 2014;30:923–30.

14. Chen Y, Chen L, Lun ATL, Baldoni PL, Smyth GK. edgeR v4: powerful differential analysis of sequencing data with expanded functionality and improved support for small counts and larger datasets. *Nucleic Acids Res* 2025;53:gkaf018.

15. Leek JT, Johnson WE, Parker HS, Jaffe AE, Storey JD. The sva package for removing batch effects and other unwanted variation in high-throughput experiments. *Bioinformatics* 2012;28:882–3.

16. Kuleshov MV, Jones MR, Rouillard AD, Fernandez NF, Duan Q, Wang Z, Koplev S, Jenkins SL, Jagodnik KM, Lachmann A, McDermott MG, Monteiro CD, et al. Enrichr: a comprehensive gene set enrichment analysis web server 2016 update. *Nucleic Acids Res* 2016;44:W90–7.

17. Milacic M, Beavers D, Conley P, Gong C, Gillespie M, Griss J, Haw R, Jassal B, Matthews L, May B, Petryszak R, Ragueneau E, et al. The Reactome Pathway Knowledgebase 2024. *Nucleic Acids Res* 2024;52:D672–8.

18. Agrawal A, Balcı H, Hanspers K, Coort SL, Martens M, Slenter DN, Ehrhart F, Digles D, Waagmeester A, Wassink I, Abbassi-Daloii T, Lopes EN, et al. WikiPathways 2024: next generation pathway database. *Nucleic Acids Res* 2024;52:D679–89.

19. Huang R, Grishagin I, Wang Y, Zhao T, Greene J, Obenauer JC, Ngan D, Nguyen D-T, Guha R, Jadhav A, Southall N, Simeonov A, et al. The NCATS BioPlanet – An Integrated Platform for Exploring the Universe of Cellular Signaling Pathways for Toxicology, Systems Biology, and Chemical Genomics. *Front Pharmacol* 2019;10:445.

20. Keenan AB, Torre D, Lachmann A, Leong AK, Wojciechowicz ML, Utti V, Jagodnik KM, Kropiwnicki E, Wang Z, Ma'ayan A. ChEA3: transcription factor enrichment analysis by orthogonal omics integration. *Nucleic Acids Res* 2019;47:W212–24.

21. Langfelder P, Horvath S. WGCNA: an R package for weighted correlation network analysis. *BMC Bioinformatics* 2008;9:559.

22. Ritchie SC, Watts S, Fearnley LG, Holt KE, Abraham G, Inouye M. A Scalable Permutation Approach Reveals Replication and Preservation Patterns of Network Modules in Large Datasets. *Cell Syst* 2016;3:71–82.

23. Szklarczyk D, Morris JH, Cook H, Kuhn M, Wyder S, Simonovic M, Santos A, Doncheva NT, Roth A, Bork P, Jensen LJ, von Mering C. The STRING database in 2017: quality-controlled protein–protein association networks, made broadly accessible. *Nucleic Acids Res* 2017;45:D362–8.

24. Ju W, Li J, Yu W, Zhang R. iGraph: an incremental data processing system for dynamic graph. *Front Comput Sci* 2016;10:462–76.

25. R core team. R [Internet]. Available from: https://cran.r-project.org/

26. Zheng Y, Luo H, Teng X, Hao X, Yan X, Tang Y, Zhang W, Wang Y, Zhang P, Li Y, Zhao Y, Chen R, et al. NPInter v5.0: ncRNA interaction database in a new era. *Nucleic Acids Res* 2023;51:D232–9.

27. Zhao H, Yin X, Xu H, Liu K, Liu W, Wang L, Zhang C, Bo L, Lan X, Lin S, Feng K, Ning S, et al. LncTarD 2.0: an updated comprehensive database for experimentally-supported functional lncRNA–target regulations in human diseases. *Nucleic Acids Res* 2023;51:D199–207.
